# Rodent monocyte‐derived macrophages do not express CD163: Comparative analysis using macrophages from living boreoeutherians

**DOI:** 10.1002/dvdy.70036

**Published:** 2025-05-12

**Authors:** Yoichi Saito, Yukio Fujiwara, Yasuka L. Yamaguchi, Satomi S. Tanaka, Kyoko Miura, Yoshiyuki Hizukuri, Kyoko Yamashiro, Yasuhiro Hayashi, Yuta Nakashima, Yoshihiro Komohara

**Affiliations:** ^1^ Laboratory of Bioengineering, Faculty of Advanced Science and Technology Kumamoto University Kumamoto Japan; ^2^ Department of Cell Pathology, Faculty of Life Sciences Kumamoto University Kumamoto Japan; ^3^ Laboratory of Molecular Embryology, Faculty of Health Science Kumamoto Health Science University Kumamoto Japan; ^4^ Department of Aging and Longevity Research, Faculty of Life Sciences Kumamoto University Kumamoto Japan; ^5^ Center for Metabolic Regulation of Healthy Aging, Faculty of Life Sciences Kumamoto University Kumamoto Japan; ^6^ Asubio Pharma Co., Ltd. Kobe Japan; ^7^ Institute of Industrial Nanomaterials Kumamoto University Kumamoto Japan; ^8^ International Research Organization for Advanced Science and Technology Kumamoto University Kumamoto Japan; ^9^ Fusion Oriented Research for Disruptive Science and Technology, Japan Science and Technology Agency Saitama Japan

**Keywords:** fetal liver, Kupffer cell, resident macrophage, tumor‐associated macrophage, yolk sac

## Abstract

**Background:**

CD163 is a scavenger receptor predominantly expressed on the surfaces of macrophages in various mammalian species and is a marker of anti‐inflammatory (M2‐like) macrophages. High density of CD163‐positive tumor‐associated macrophages (TAMs) is associated with worse prognosis in various patient tumors. Interestingly, studies on mice have shown that CD163‐positive TAMs only infiltrate the margins of tumor tissues, not the center. Based on these observations, we hypothesized that circulating monocyte‐derived macrophages (MDMs), which are the origin of most TAMs, do not express CD163 in mice.

**Results:**

We examined CD163 expression in MDMs, differentiated from healthy animals in vitro, and in normal, pathogenic, and tumorigenic macrophages infiltrating various tumors and organs across multiple species including primates, rodents, cetartiodactylans, and carnivores. We found that MDMs, including TAMs, do not express CD163 in mice. Our findings also suggest that murine CD163‐positive macrophages likely originate from a specific subset of resident macrophages, namely fetal liver monocytes/macrophages, as indicated by fetal analysis. Furthermore, we revealed that the CD163‐negative expression pattern in MDMs is a trait shared by the rodent clade.

**Conclusions:**

Rodent MDMs do not express CD163, a phenotype not shared with MDMs of other mammals. Our findings caution against the extrapolation of rodent experimental results to other animal models.

## Key Findings

- We investigated CD163 expression in monocyte‐derived macrophages (MDMs) from healthy animals in vitro and in macrophages from various tumors and organs across primates, rodents, cetartiodactyls, and carnivores. We found that MDMs, including tumor‐associated macrophages, do not express CD163 in mice. Our fetal mouse analysis suggests that CD163‐positive macrophages are derived from resident macrophages, specifically fetal liver monocytes/macrophages. Additionally, the lack of CD163 expression in rodent MDMs appears to be a rodent‐specific trait that is not shared with other mammals. It highlights the need for caution when extrapolating rodent experimental results to other animal models.

## 1 INTRODUCTION

Macrophages are broadly classified into two functional types: classically activated macrophages (M1‐like macrophages), which induce inflammation, and alternatively activated macrophages (M2‐like macrophages), which perform various functions in anti‐inflammatory responses, tissue regeneration, and repair.^1^, ^2^ Studies involving humans and mice have shown that M1‐like macrophages produce proinflammatory cytokines that stimulate antitumor immune responses, while M2‐like macrophages promote angiogenesis, immunosuppression, and tumor progression by secreting growth‐promoting molecules.^3^ Consistently, research on various inflammatory diseases has demonstrated that M1‐like macrophages exacerbate and prolong inflammatory reactions, while M2‐like macrophages contribute to immunosuppression, tissue repair, and organogenesis.^4^, ^5^ Hence, surface markers that reflect the activation state of macrophages have been studied as both exacerbating and prognostic factors for a range of diseases.^6^

CD163 is a scavenger receptor predominantly expressed on the surfaces of macrophages across different mammalian species.^7^, ^8^ It binds various ligands, including the hemoglobin/haptoglobin complex, bacteria, viruses, and the tumor necrosis factor‐like weak inducer of apoptosis (TWEAK).^9^, ^10^, ^11^, ^12^ CD163 expression in human monocytes/macrophages is induced by interleukin (IL)‐10 or dexamethasone.^13^, ^14^, ^15^ CD163‐positive macrophages have been shown to play significant roles in malignant tumors, autoimmune diseases, and lung diseases, and CD163 is considered a M2‐like macrophage marker. For example, in inflammatory liver diseases, CD163‐negative macrophages are strongly associated with the progression of liver fibrosis.^16^, ^17^ On the other hand, tumor‐associated macrophages (TAMs) exhibit phenotypic heterogeneity, and a high density of CD163‐positive TAMs is associated with a worse prognosis in many malignant tumors in humans.^18^, ^19^, ^20^, ^21^, ^22^, ^23^, ^24^, ^25^, ^26^ CD163‐positive TAMs have also been reported to be correlated with tumor grade in canine and feline malignancies.^27^, ^28^, ^29^ However, there are few reports on the same in mouse malignancies that are based on firm histological analysis. Therefore, in our previous study, using a subcutaneous transplantation model of mouse sarcoma cell lines into CD163 knockout (KO) and wild‐type (WT) mice, we found that WT mice had significantly larger sarcomas, more incidence of lung metastasis, and shorter survival times than CD163KO mice.^24^ Interestingly, CD163‐positive TAMs only infiltrated the margins of the sarcoma tissues and not the centers. In another study, we performed subcutaneous implantation of fibrin hydrogels in mice to promote tissue regeneration by infiltrating M2‐like macrophages; however, CD163‐positive macrophages were present only around the gel, and those infiltrating the gel were CD163‐negative.^30^ The origin of macrophages can be broadly divided into embryonic and monocyte‐derived. In adults, the majority of macrophages newly infiltrating tumors, wounds, and inflammatory sites are derived from monocytes.^31^, ^32^, ^33^ Based on these observations and consensus, we hypothesized that monocyte‐derived macrophages do not express CD163 in mice.

Based on this hypothesis, in the present study, we examined CD163 expression in TAMs, as well as normal, pathogenic, and tumorigenic macrophages infiltrating various tumors and organs of multiple species of primates, rodents, and cetartiodactylans (livestock species). We also analyzed CD163 expression in monocyte‐derived macrophages from similar species and bone marrow‐derived and peritoneal macrophages from mice. Particularly, our analysis focused on the cellular origin of murine macrophages, utilizing fetal samples and employing multiple disease models and analytical methods from various perspectives. We further discussed these results phylogenetically, referring to previous literature on mammals, including carnivores (pet species).

## 2 RESULTS

### 2.1 Differential CD163 expression in human and murine TAMs

**FIGURE 1:**
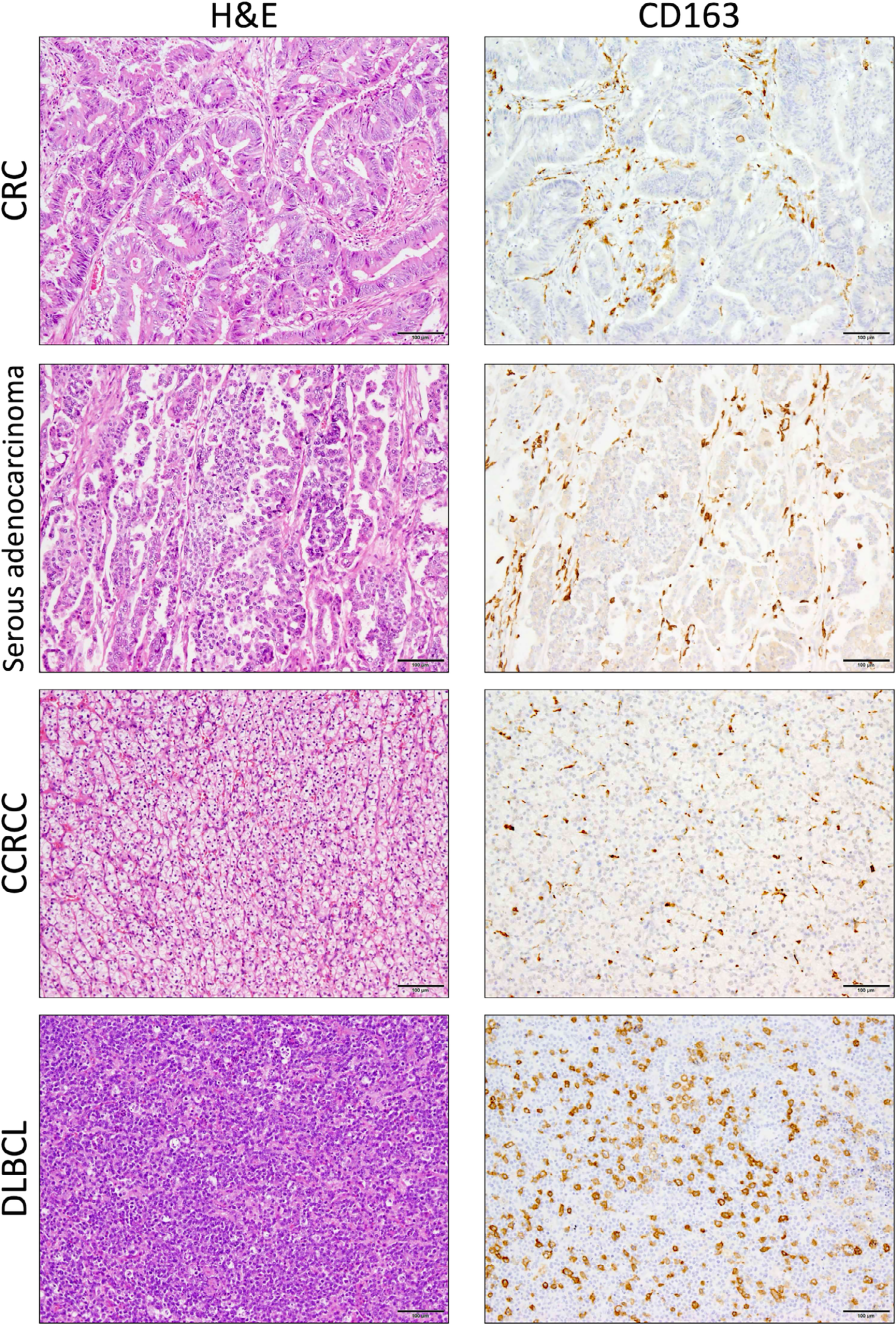
Typical CD163 expression in various human clinical malignant tumor specimens. Hematoxylin and eosin (H&E) and immunohistochemical staining showing CD163 in cases of colorectal cancer (CRC), ovarian serous adenocarcinoma, clear cell renal cell carcinoma (CCRCC), and diffuse large B‐cell lymphoma (DLBCL). Scale bars: 100 μm.

Several types of human malignant tumor tissue samples, including colorectal cancer, ovarian serous adenocarcinoma, clear cell renal cell carcinoma, and diffuse large B cell lymphoma, were collected and immunostained for CD163 (Figure 1). Numerous CD163^+^ TAMs were observed spreading from the middle to the edges of the tumor tissues.

**FIGURE 2:**
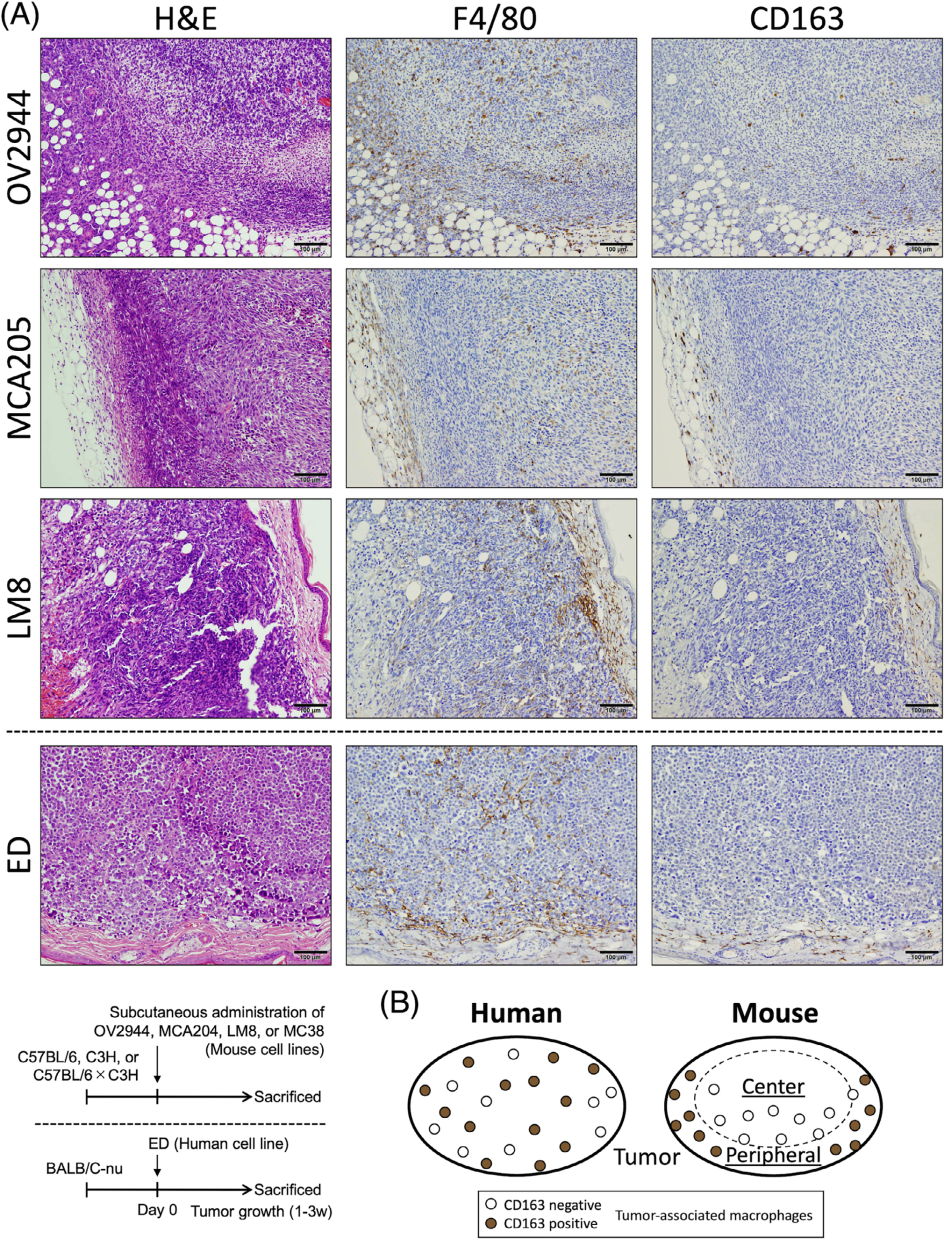
CD163 expression in various cell line tumors subcutaneously injected into mice. (A) H&E and immunohistochemical staining of F4/80 and CD163 in OV2944, MCA205, LM8, and ED cell lines. Scale bars: 100 μm. (B) Schematic representation of differences in CD163 expression between human and mouse tumors. CD163‐positive tumor‐associated macrophages (TAMs) are diffusely distributed in human tumors (left circle). In contrast, CD163‐positive TAMs are not centrally distributed in mouse tumors (right circle).

Next, we conducted similar experiments with tumor‐bearing mice. To this end, three mouse cancer cell lines (OV2944; ovarian cancer from C57BL/6×C3H, MCA205; fibrosarcoma from C57BL/6, and LM8; osteosarcoma from C3H) were injected subcutaneously into strain‐matched mice. A human leukemia cell line (ED) was injected subcutaneously into immunodeficient Balb/c nude mice as a xenograft model. After the tumors grew, they were removed and immunostained to look for two markers, F4/80 and CD163 (Figure 2A). F4/80^+^ TAMs infiltrated the entire tumor of each cell line, while CD163^+^ TAMs were present in small numbers only at the margins of each tumor. This histology was also observed in ED xenografted Balb/c nude mice because the host mouse macrophages were intervening as TAMs. Thus, these findings suggest that, unlike human TAMs, murine TAMs may not express CD163 (Figure 2B).

### 2.2 CD163 is not expressed on murine MDMs and BMDMs

**FIGURE 3:**
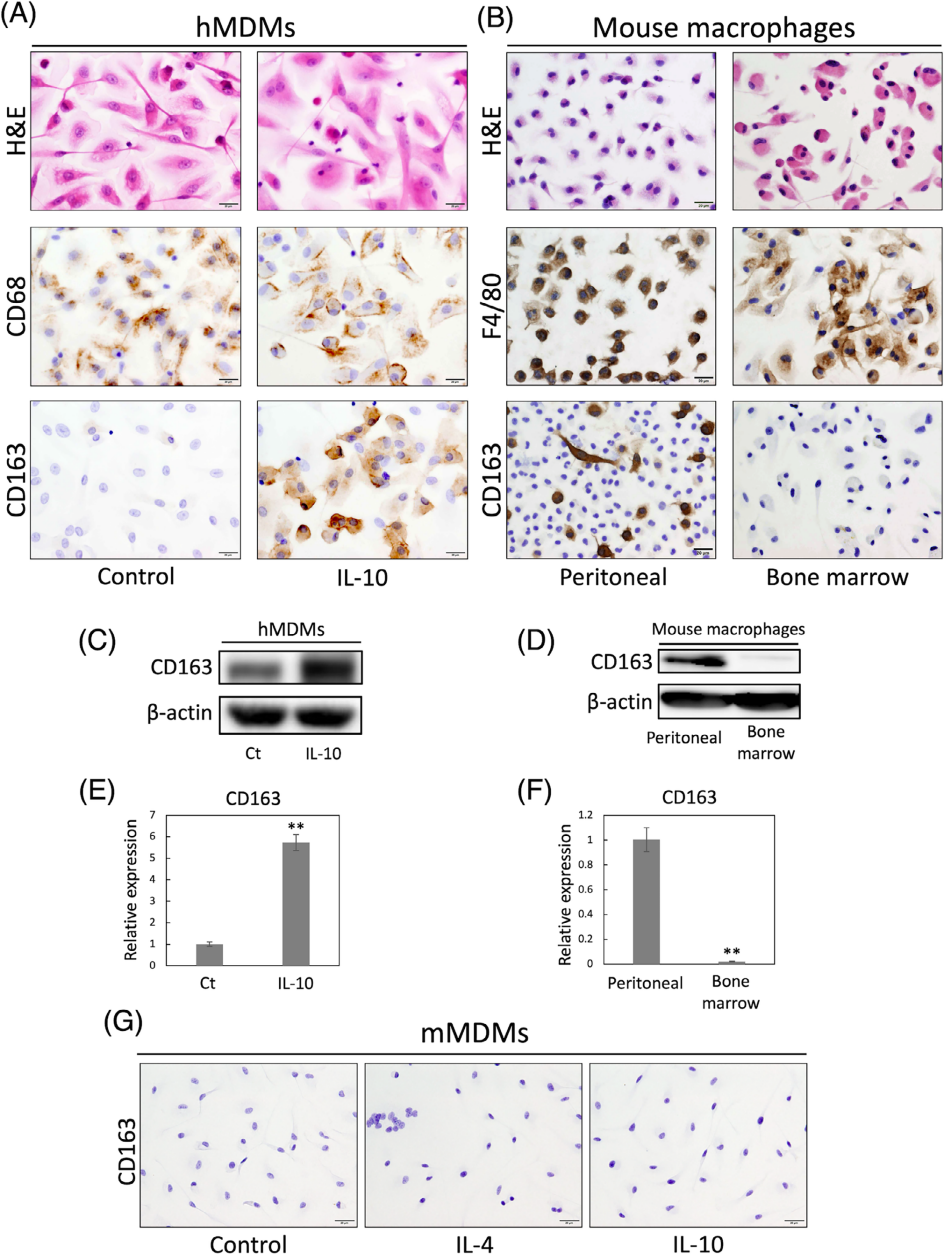
Differential CD163 expression between human and mouse macrophages in vitro. (A) H&E and immunocytochemical staining of CD68 and CD163 in human monocyte‐derived macrophages (MDMs) stimulated with interleukin (IL)‐10 during the last 24 h of culture, compared with untreated control macrophages. Scale bars: 20 μm. (B) H&E and immunocytochemical staining of F4/80 and CD163 in mouse peritoneal and bone marrow‐derived macrophages. Scale bars: 20 μm. (C,D). Western blotting analyses of CD163 in macrophages as described in A and B. (E,F) *CD163* gene expression of macrophages as described in A and B. Data are presented as the mean ± standard deviation (SD). **, *p* < .01. (G) Immunocytochemical staining of CD163 in mouse monocyte‐derived macrophages (mMDMs) stimulated with IL‐4 or IL‐10 during the last 24 h of culture, compared with untreated control macrophages. Scale bars: 20 μm.

Given that most TAMs are derived from tumor‐infiltrating monocytes, we assessed CD163 mRNA and protein expression on human monocyte‐derived macrophages (hMDMs) and murine macrophages in vitro by immunocytochemistry (Figure 3A,B), western blotting (Figure 3C,D) and qPCR (Figure 3E,F). CD163 was expressed on hMDMs, and the expression was enhanced by IL‐10 stimulation (Figure 3A,C,E). On the other hand, CD163 expression was not observed in murine bone marrow‐derived macrophages (mBMDMs), while a population of CD163^+^ macrophages existed in murine peritoneal macrophages (Figure 3B,D,F). Additionally, CD163 expression was not observed in murine monocyte‐derived macrophages (mMDMs) on stimulation with IL‐4 or IL‐10 (Figure 3G), suggesting that CD163 is perhaps expressed on murine peritoneal macrophages, whereas mMDMs, unlike hMDMs, do not express CD163.

### 2.3 CD163 gene expression is low in mBMDMs regardless of activation state

**FIGURE 4:**
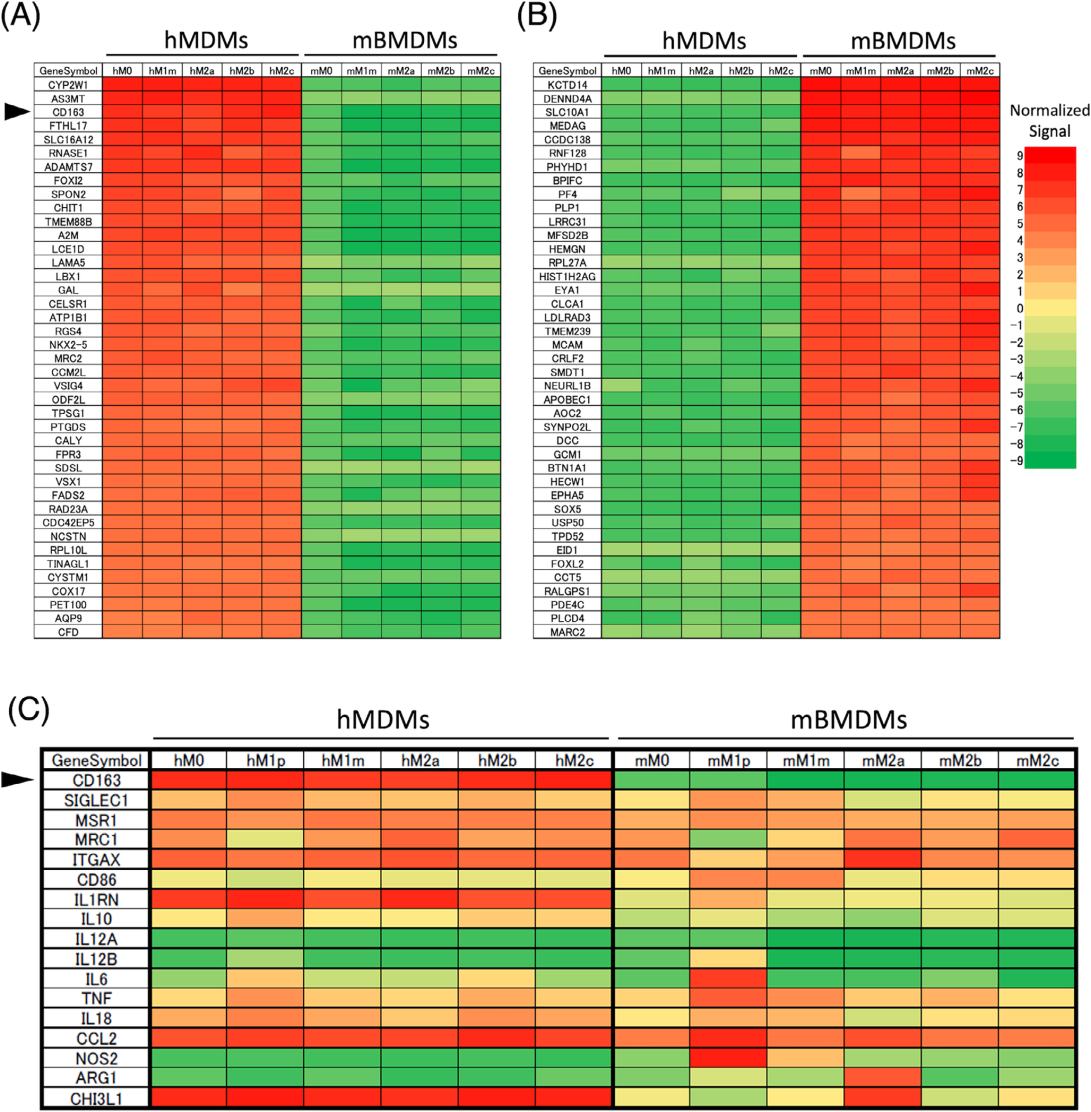
Heatmaps of macrophage‐specific gene expression in hMDMs and mBMDMs. (A) hMDM‐specific genes; normalized signal of >4 and <−3 across all hMDMs and mBMDMs subtypes, respectively. (B) mBMDM‐specific genes; normalized signal of <−3 and >4 across all hMDMs and mBMDMs subtypes, respectively. Extracted human and mouse macrophage‐specific genes were sorted in descending order of human and mouse M0, respectively, and the top 41 genes are represented. (C) Representative genes of macrophage surface markers, cytokines, and transcription factors extracted from the heatmap. Red color indicates higher expression, and green color indicates lower expression. Arrowheads indicate CD163.

CD163 is an M2 phenotype marker in mice as well as humans, although reported relatively infrequently.^24^, ^34^, ^35^, ^36^ We activated both human and mouse macrophages (hMDMs and mBMDMs) using different stimuli, and then determined the gene expression of several macrophage activation markers using a microarray. Genes that were strongly expressed in either hMDMs or mBMDMs and not in the other, regardless of stimulus, were shown in Figure 4A,B. At this point, CD163 was classified into a group that is hardly expressed in mBMDMs (Figure 4A). Then, CD163 and representative macrophage markers were extracted in Figure 4C. For example, NOS2 and ARG1 were only expressed on specific subsets of activated macrophages in mice, M1p and M2a, respectively. On the other hand, SIGLEC1 (CD169), MRC1 (CD206), TNF, and IL6 showed similar expression patterns in both humans and mice. SIGLEC1 (CD169) and MRC1 (CD206) and IL10 were generally more strongly expressed in humans. CD86, TNF, and IL6 were strongly expressed in M1 selectively in mice. Another representative macrophage surface marker, MSR1 (CD204) was expressed in both types of macrophages and in both species. CD163, IL1RN (IL‐1Ra), and CHI3L1 (YKL‐40) were particularly strongly expressed in hMDMs. However, CD163 expression was notably low in mBMDMs, regardless of the type of stimulation (Figure 4A,C). These findings suggest that mouse macrophages derived from bone marrow monocytes may not naturally express CD163.

### 2.4 Differential CD163 expression in liver resident macrophages (Kupffer cells) and hepatoma‐infiltrating TAMs

**FIGURE 5:**
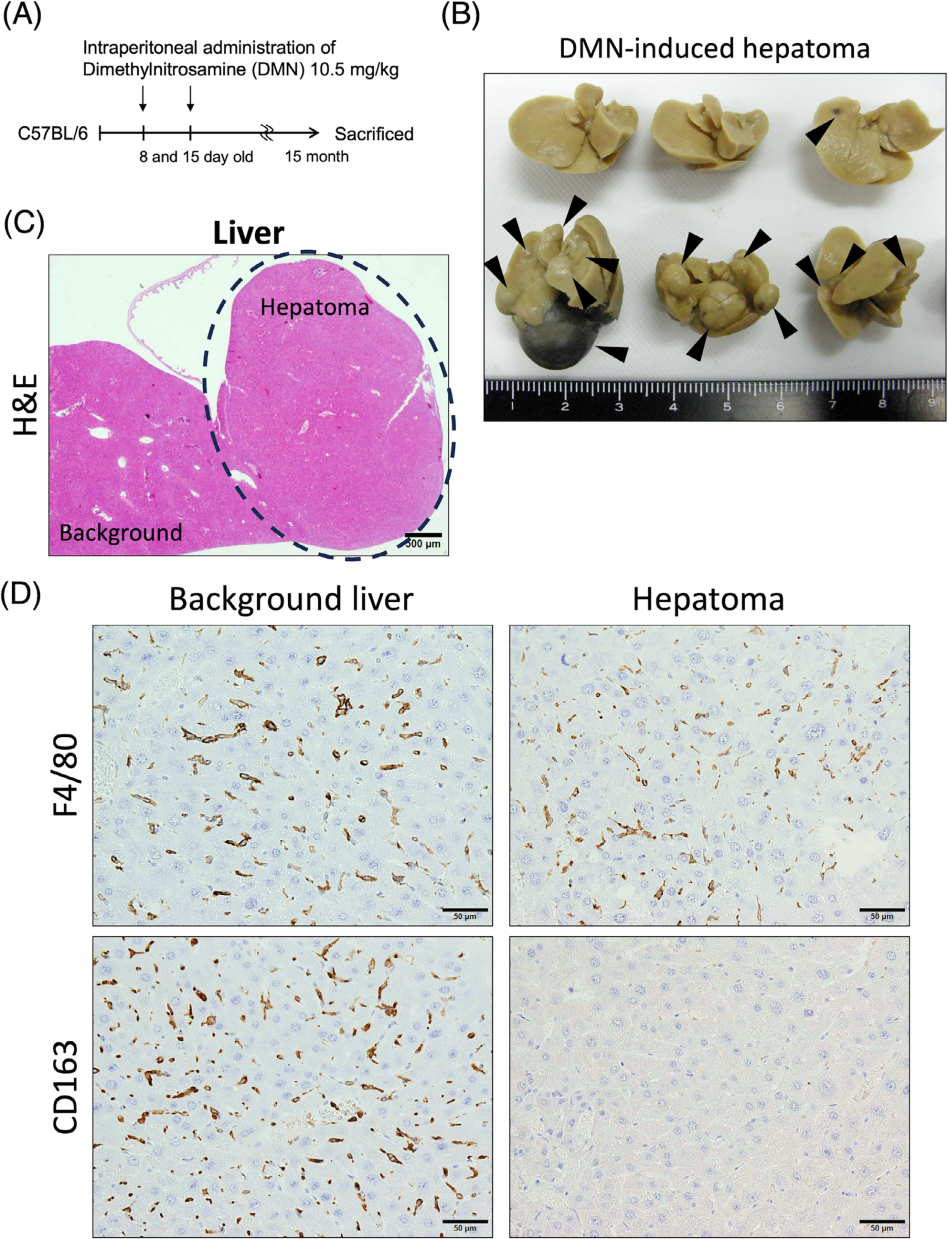
CD163 expression in dimethylnitrosamine (DMN)‐induced hepatoma. (A) Protocol for DMN administration in C57BL/6 mice. (B) Macroscopic image of the liver after 15 months of DMN administration. Arrowheads indicate hepatomas. Scale units: Cm. (C) H&E staining of the liver with hepatoma. A dashed line encloses the hepatoma area. Scale bar: 100 μm. (D) Immunohistochemical staining of F4/80 and CD163 in the background liver and hepatoma. Scale bars: 50 μm.

Numerous tissue‐resident macrophages called Kupffer cells are localized in the sinusoids of the liver. In the present study, we used a dimethylnitrosamine (DMN)‐induced hepatoma model to compare CD163 expression between hepatoma‐infiltrating TAMs and Kupffer cells as resident macrophages. Fifteen months after intraperitoneal injection of DMN, multiple liver tumors were observed in the liver of several mice (Figure 5A,B). Immunohistochemical analysis revealed that Kupffer cells localized to the background liver were CD163^+^/F4/80^+^ cells; however, hepatoma‐infiltrating TAMs were CD163^−^/F4/80^+^ cells (Figure 5C,D).

**FIGURE 6:**
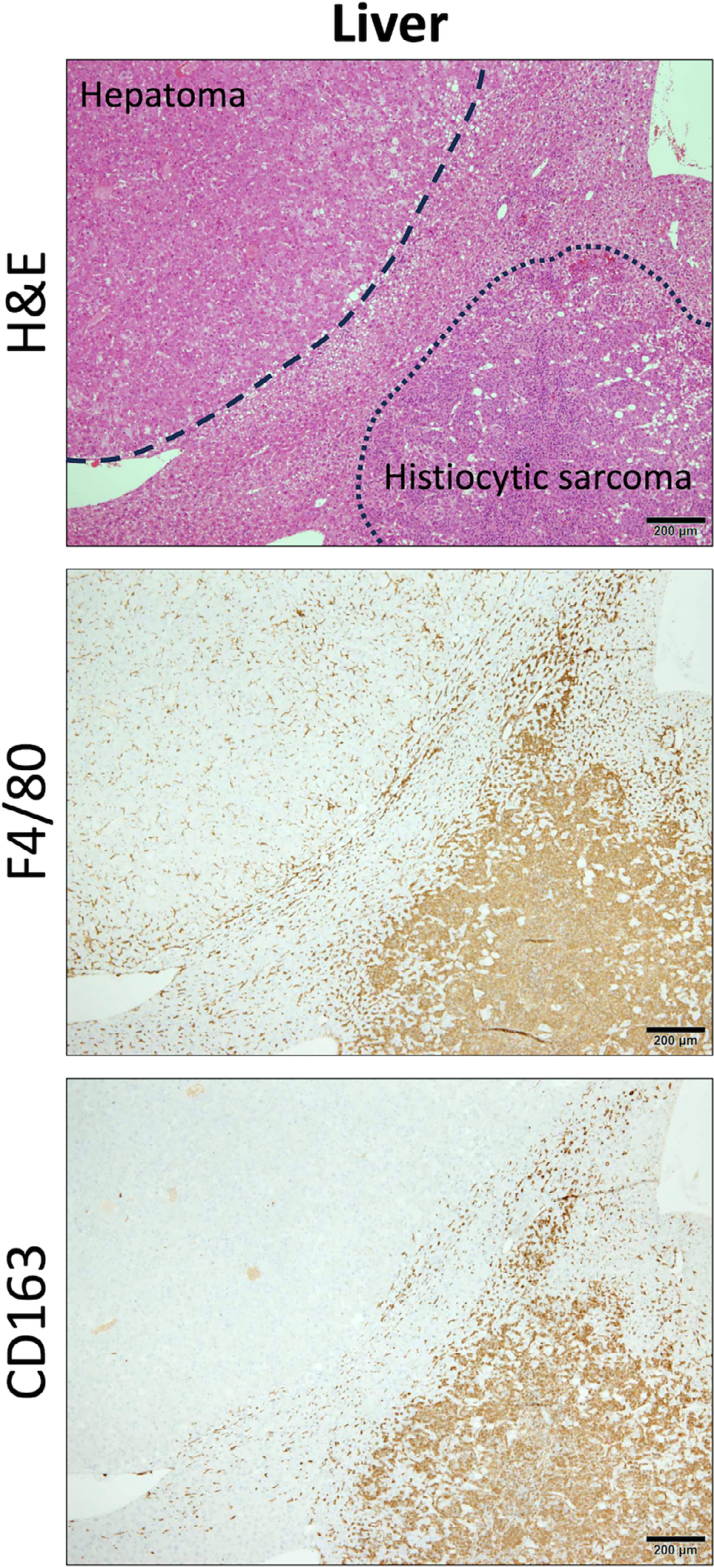
CD163 expression in spontaneous hepatoma and histiocytic sarcoma. H&E and immunohistochemical staining of F4/80 and CD163 in the liver with hepatoma and histiocytic sarcoma. Dashed and dotted lines enclose the hepatoma and histiocytic sarcoma areas, respectively. Scale bars: 200 μm.

**FIGURE 7:**
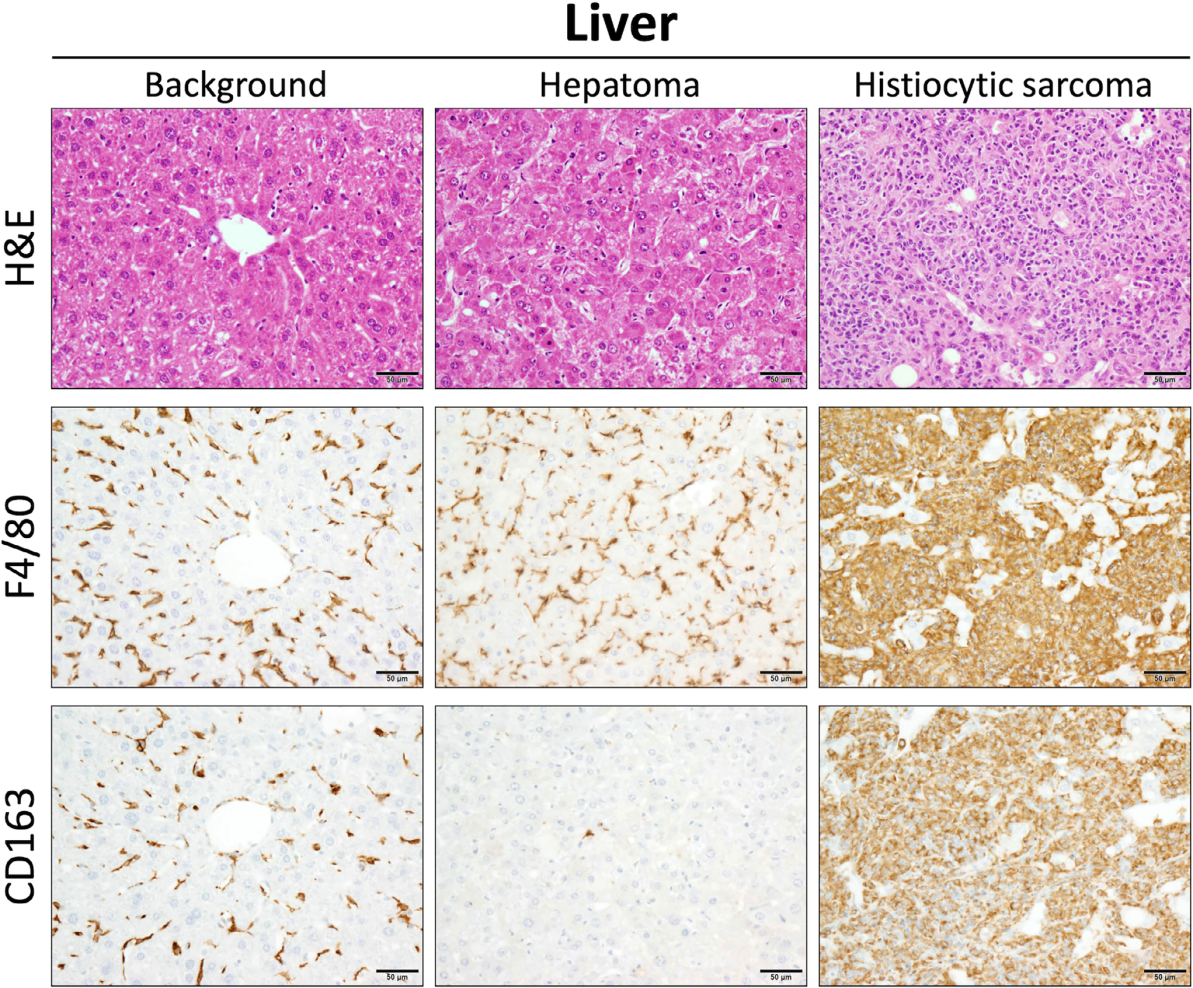
Immunohistological comparison of macrophages in background liver, hepatoma, and histiocytic sarcoma. High magnification images of H&E and immunohistochemical staining of F4/80 and CD163 in the background liver, hepatoma, and histiocytic sarcoma. Scale bars: 50 μm.

**FIGURE 8:**
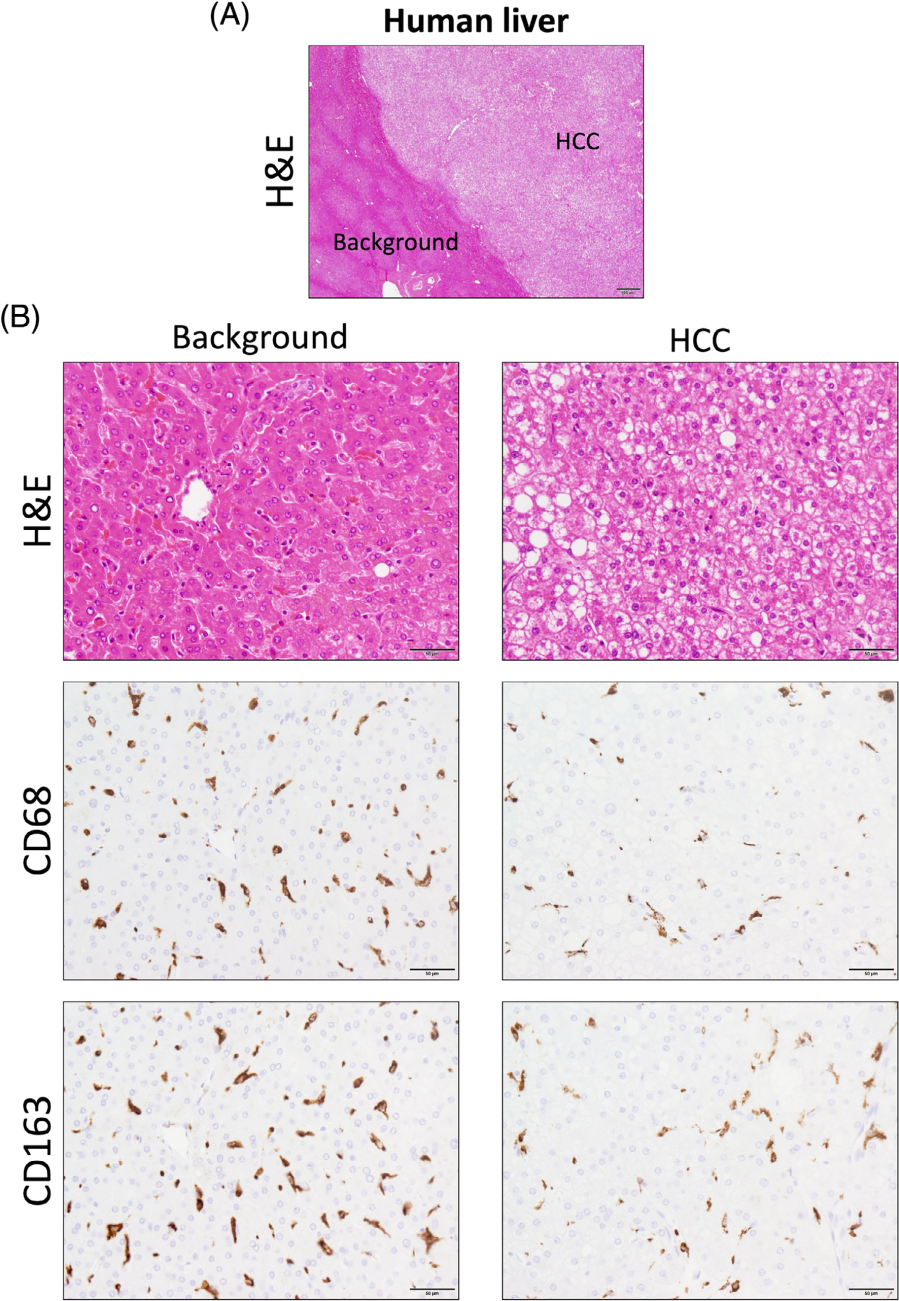
CD163 expression in hepatocellular carcinoma (HCC) in human clinical specimens. (A) H&E staining of a human liver specimen with HCC. Scale bar: 500 μm. (B) H&E and immunohistochemical staining of CD68 and CD163 in the background liver and HCC. Scale bars: 50 μm.

We also investigated a tissue sample from a mouse that spontaneously developed two types of tumors, histiocytic sarcoma and hepatoma (Figure 6). TAMs infiltrating spontaneous hepatomas were negative for CD163 (Figure 7). However, the histiocytic sarcoma cells, which may be tumorigenic Kupffer cells, were positive for CD163, similar to Kupffer cells in the background liver. In contrast, TAMs infiltrating hepatocellular carcinoma (HCC) in humans were positive for CD163, the same as that observed in other human tumors (Figure 8). These findings suggest that mouse macrophages derived from bone marrow or peripheral blood monocytes do not naturally express CD163.

### 2.5 CD163 ^−^/CD206 ^+^ TAMs are present in murine tumors

**FIGURE 9:**
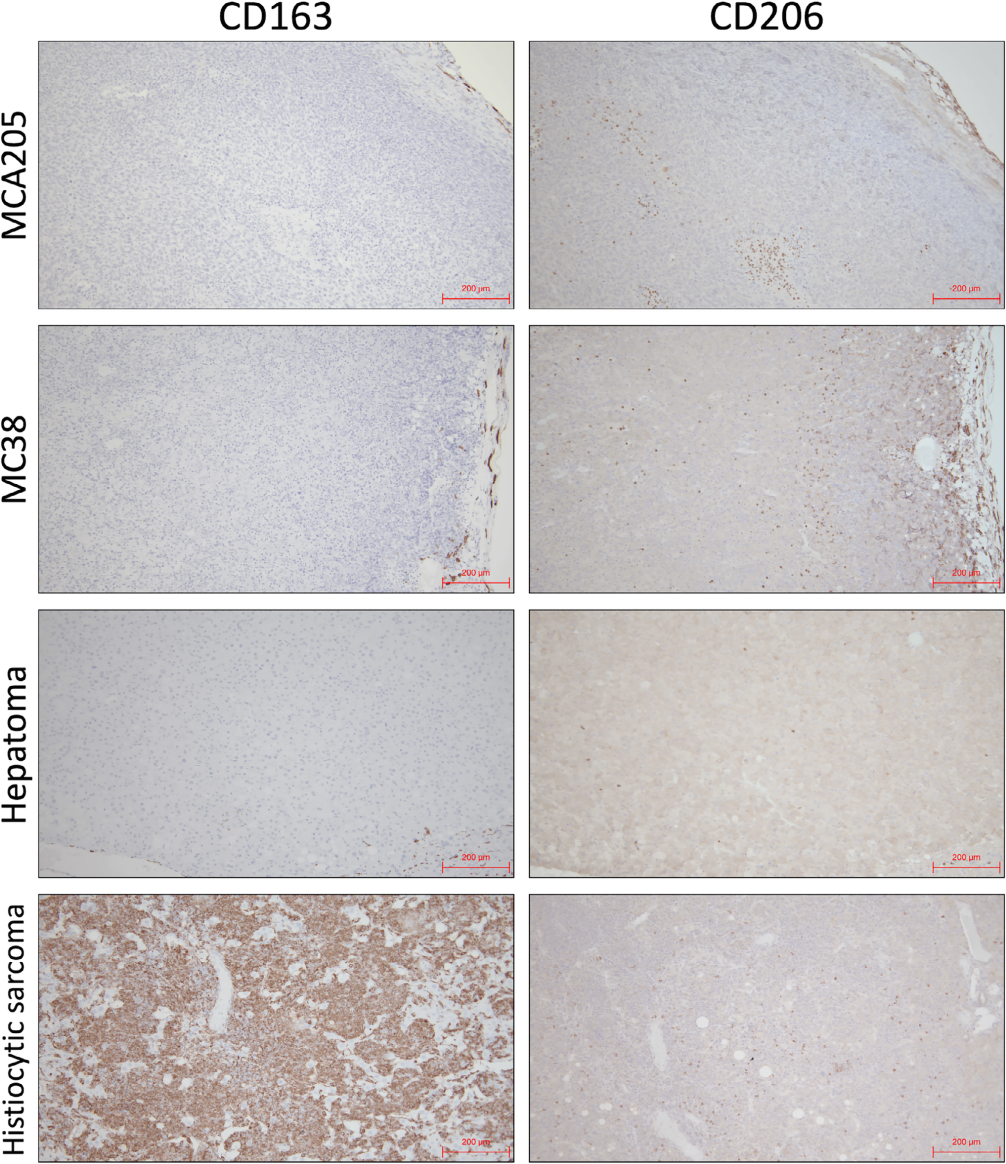
Immunohistological comparison of CD163 and CD206 expression in various murine tumors. Immunohistochemical staining of CD163 and CD206 in MCA205, MC38, hepatoma, and histiocytic sarcoma. Scale bars: 200 μm.

To analyze the expression of M2 macrophage surface markers other than CD163 in TAMs, immunostaining for CD206 was performed. CD206‐positive cells infiltrated within the various tumors, albeit in small numbers (Figure 9). These cells were CD163^−^/CD206^+^ TAMs because none of the CD163‐positive cells were found at the same site, with the exception of histiocytic sarcoma. The presence of CD163^−^/CD206^+^ macrophages was consistent with the microarray results (Figure 4C). These findings suggest that mouse TAMs do not express CD163 regardless of the activation environment in which they are located.

### 2.6 CD163 is not expressed in recruited macrophages after depletion by clodronate liposome

**FIGURE 10:**
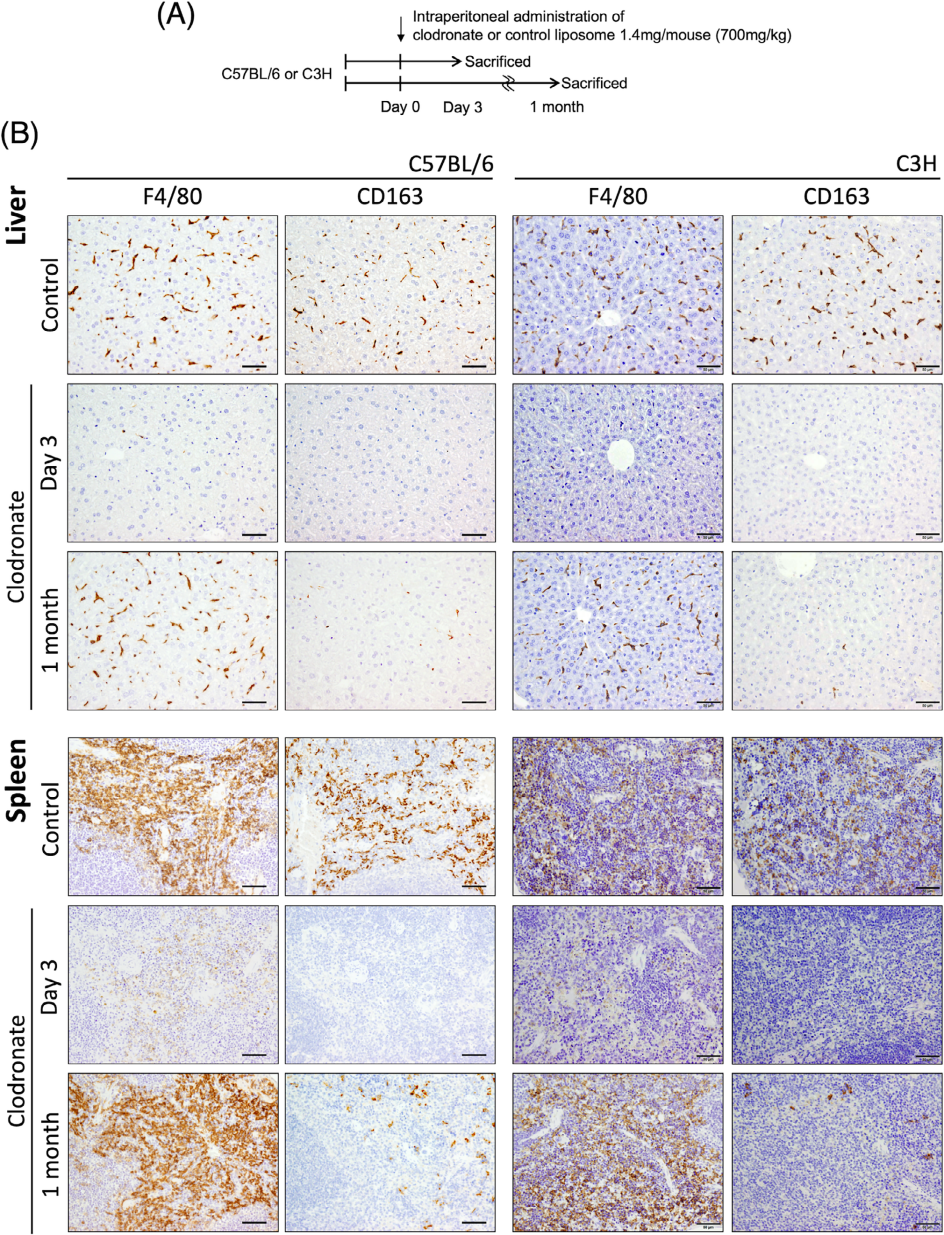
Immunohistological comparison of macrophages after clodronate liposome‐mediated macrophage depletion. (A) Protocol for clodronate liposome administration in C57BL/6 and C3H mice. (B) Immunohistochemical staining of F4/80 and CD163 in the liver and spleen 3 days and 1 month post‐clodronate administration to C57BL/6 and C3H mice. Scale bars: 50 μm.

**FIGURE 11:**
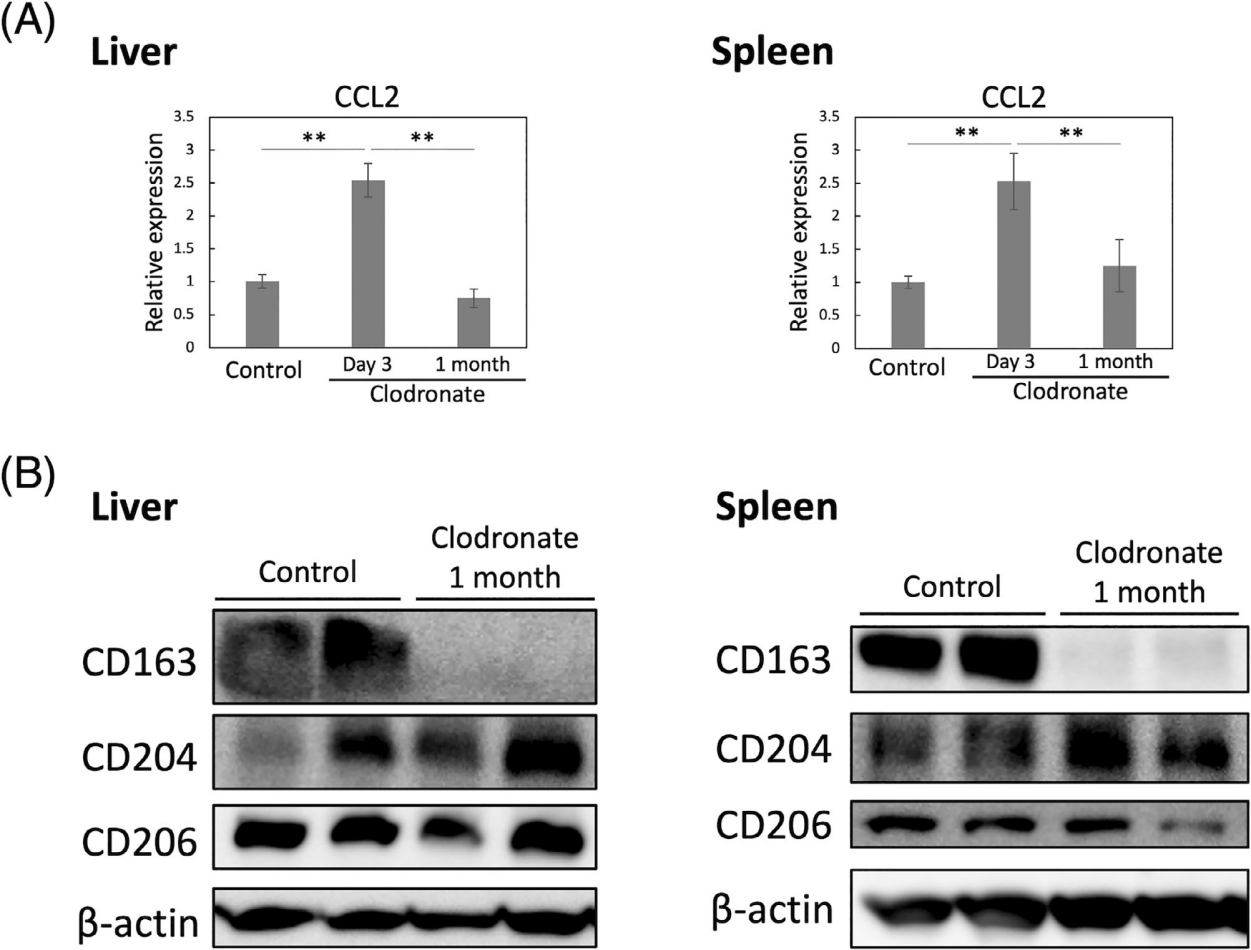
Expression of macrophage surface markers on recovered macrophages after clodronate liposome‐mediated macrophage depletion. (A) *CCL2* gene expression in the liver and spleen 3 days and 1 month post‐clodronate administration in C57BL/6 mice. Data presented as mean ± SD. **, *p* < .01. (B) Western blot analyses of CD163, CD204, CD206 in the liver and spleen 1 month post‐clodronate administration in C57BL/6 mice.

To confirm similar phenomena in non‐tumor environments, we next analyzed the liver and spleen in a clodronate liposome‐induced macrophage depletion model. Clodronate or control liposome was injected intraperitoneally into C57BL/6 and C3H mice. The liver and spleen were excised 3 days or 1 month after the injection (Figure 10A). Three days after clodronate liposome injection, nearly all macrophages in both organs were depleted when we checked for F4/80‐ and CD163‐positive macrophages by immunohistochemistry (Figure 10B). A month after clodronate liposome injection, the number of macrophages was restored; however, the majority of them did not exhibit CD163 expression. Furthermore, we found that *CCL2* mRNA expression was significantly high on day 3 post‐clodronate liposome injection compared to that at other time points (Figure 11A). This finding suggests that recovered macrophages that did not express CD163 were recruited from circulating monocytes.^37^, ^38^ Similar results were confirmed by western blotting (Figure 11B). Interestingly, there were no significant changes in the expression of other typical macrophage surface markers, CD204 and CD206, suggesting that mouse macrophages derived from bone marrow or peripheral blood monocytes do not express CD163.

### 2.7 Decreased CD163 expression in fatty liver is primarily caused by infiltration of CD163‐negative MDMs

**FIGURE 12:**
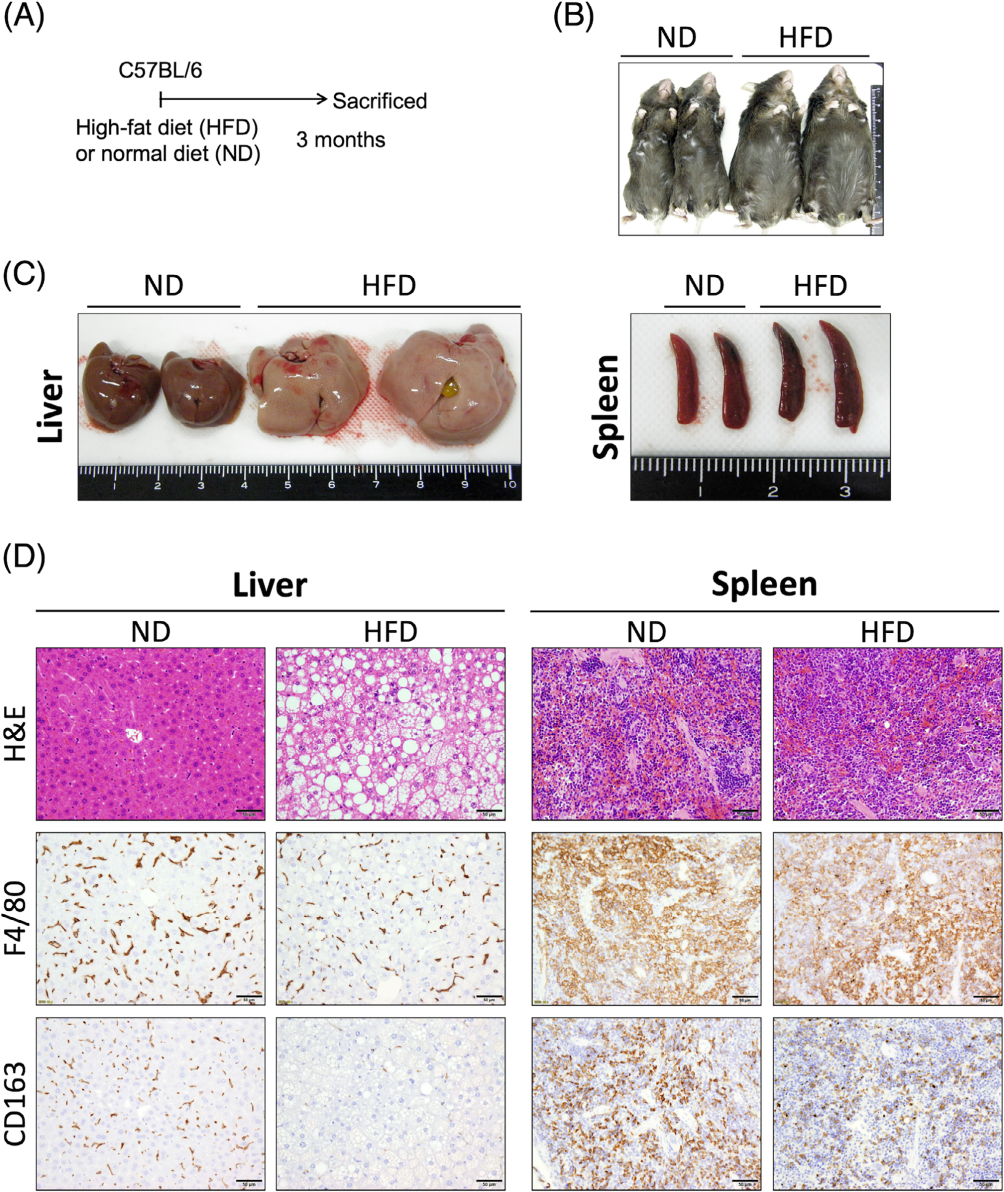
CD163 expression in liver and spleen of obese mice. (A) Protocol for administration of a high‐fat diet (HFD) or normal diet (ND) to C57BL/6 mice. (B,C) Macroscopic images of the mice (B) and their organs (C) after 3 months post‐ND or HFD administration. Scale units: Cm. (D) H&E and immunohistochemical staining of F4/80 and CD163 in the liver and spleen 3 months post‐ND or HFD administration. Scale bars: 50 μm.

**FIGURE 13:**
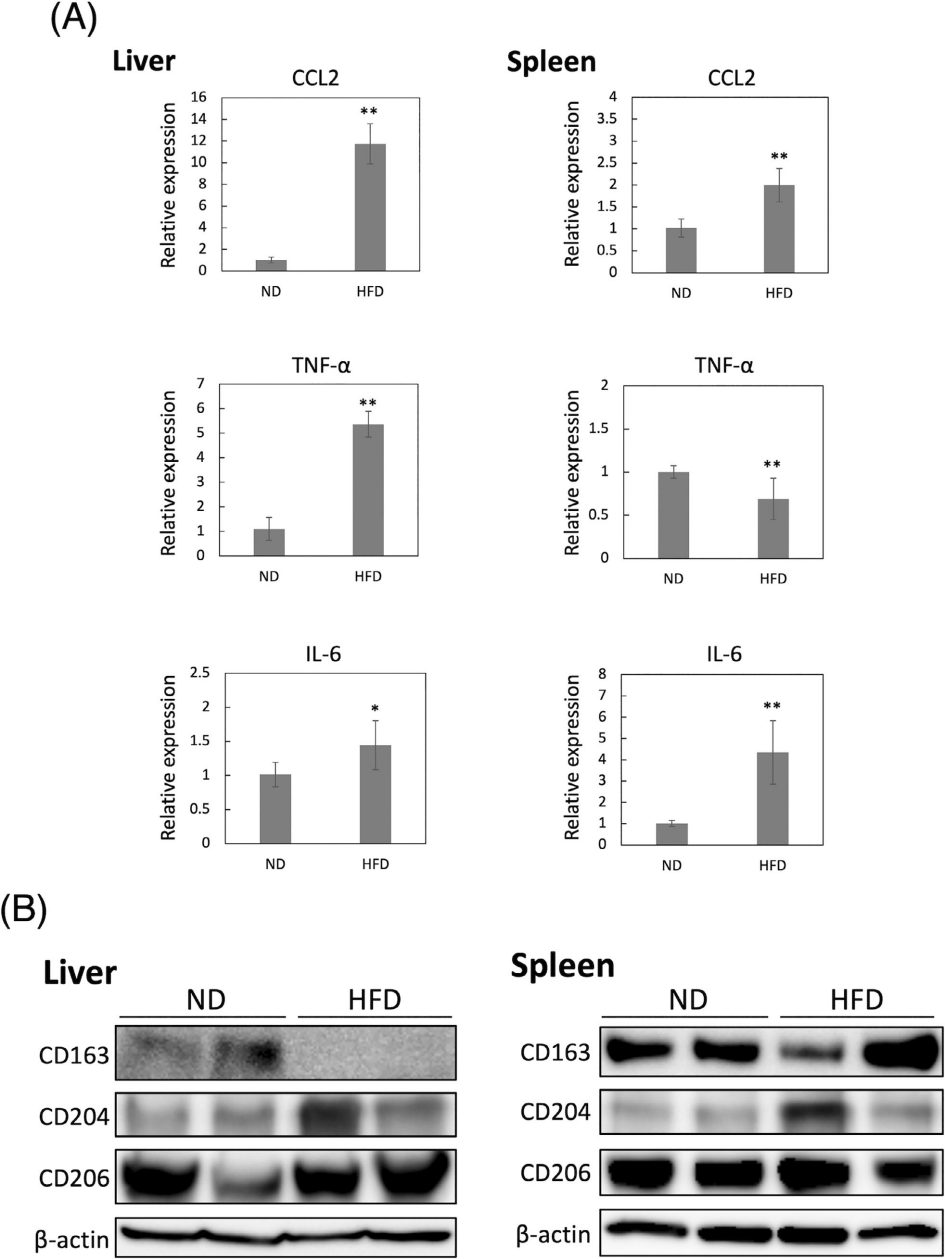
Expression of macrophage surface markers in the liver and spleen of obese mice. (A) Gene expression of *CCL2*, *TNF‐α*, and *IL‐6* in the liver and spleen 3 months post‐ND or HFD administration. Data presented as mean ± SD. *, *p* < .05. **, *p* < .01. (B) Western blot analyses of CD163, CD204, CD206 in the liver and spleen 3 months post‐ND or HFD administration.

To confirm that the same phenomena are observed in non‐neoplastic and common metabolic conditions, we assessed the liver and spleen in high‐fat diet (HFD)‐induced obese mice (Figure 12A). HFD induced obesity in mice as evidenced by weight gain (Figure 12B). Additionally, the liver became enlarged and white, which was histologically confirmed to be steatosis of hepatocytes (Figure 12C,D). The majority of the macrophages in the fatty liver were CD163‐negative macrophages (Figure 12D). In contrast, no changes in weight and histology were noted in the spleen (Figure 12C,D). Additionally, the mRNA expression of *CCL2* and inflammatory cytokines (TNF‐α and IL‐6) was much higher in the fatty livers compared to that in normal livers (Figure 13A). These findings suggest that MDMs, originally CD163‐negative macrophages, were recruited in response to inflammation because there were no significant changes in the expression of other typical macrophage surface markers, CD204 and CD206, in the liver (Figure 13B).

### 2.8 Distribution of CD163‐positive macrophages in adult mice

**FIGURE 14:**
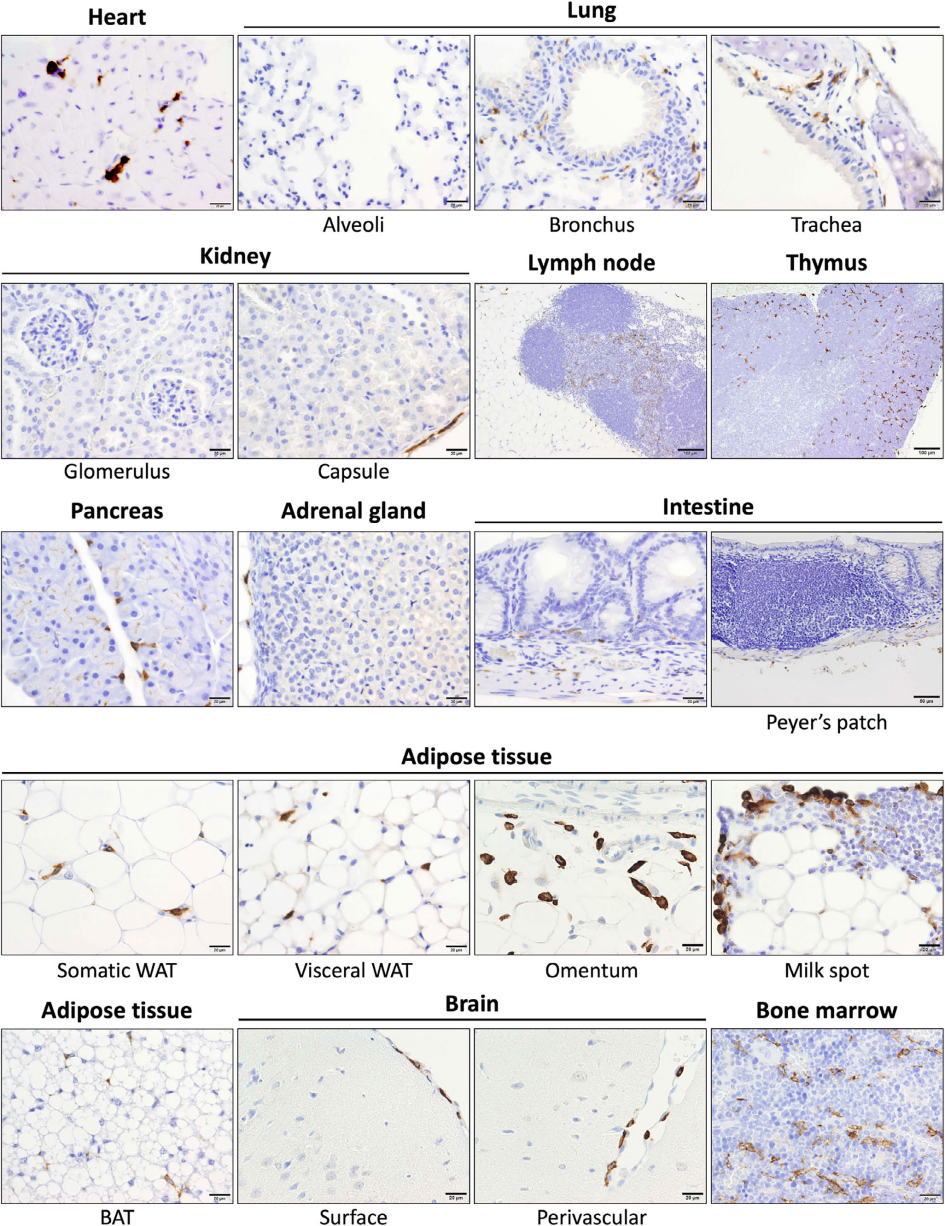
CD163 expression in organs of adult healthy C57BL/6 mice. Immunohistochemical staining of CD163 in each organ. Scale bars: 20 μm, except in the lymph node (100 μm), thymus (100 μm), and Payer's patches (50 μm). BAT, brown adipose tissue; WAT, white adipose tissue.

MDMs and tissue‐resident macrophages are distributed in various proportions in different organs. Therefore, we assessed CD163 expression in macrophages in several organs of adult healthy mice by immunostaining for the same (Figure 14). As shown in Table 1, several tissue‐specific macrophages (alveolar macrophages, Langerhans cells, microglia, osteoclasts, etc.) were CD163‐negative. These include tissue‐resident macrophages that are thought to be derived from embryonic (yolk sac) macrophages, suggesting that it is not just MDMs that do not express CD163.

**TABLE 1:** Reactivity of ICA‐TG4‐RBP1, EPR19518 with mouse macrophages (C57BL/6).

Organs and macrophages	Reactivity (positive cells)
Heart	
Intermuscular	+
Lung	
Alveolar	−
Parabronchial	+
Paratracheal	+
Liver	
Kupffer	+
In portal triads	+
Kidney	
Glomerular	−
Inerstitial	−
Capsular	+
Spleen	
Red pulp	+
White pulp	−
Lymph nodes	
In follicles	−
Extra‐follicles	+
Thymus	
Cortical	+
Medullary	−
Pancreas	
Interstitial	+
Adrenal	
Interstitial	−
Capsular	+
Intestines	
In lamina propria	+
In other layers	+
Bladder	
Interstitial	+
Skin	
Langerhans	−
Dermal	+
Adipose	
White somatic	+
White visceral	+
Omental	+
Brown	+
Skeletal muscle	
Intramuscular	+
Brain	
Microglias	−
Perivascular	+
Surface (subarachnoid)	+
In choroid plexsus	+
Bone	
Osteoclasts	−
In marrow	+

### 2.9 Distribution of CD163‐positive macrophages in fetal mice

**FIGURE 15:**
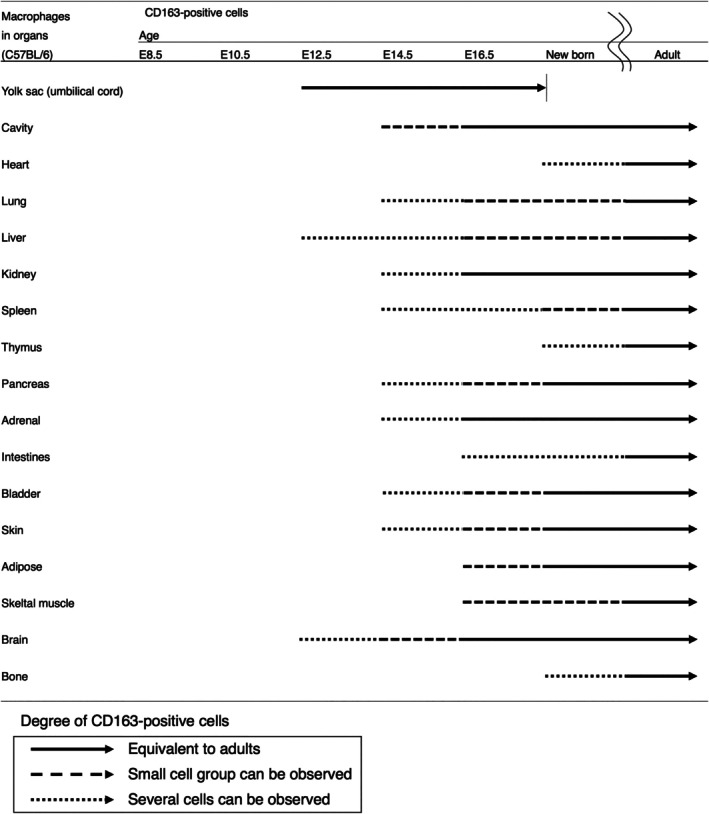
Degree of localization of CD163‐positive macrophages in different organs and embryonic ages.

**FIGURE 16:**
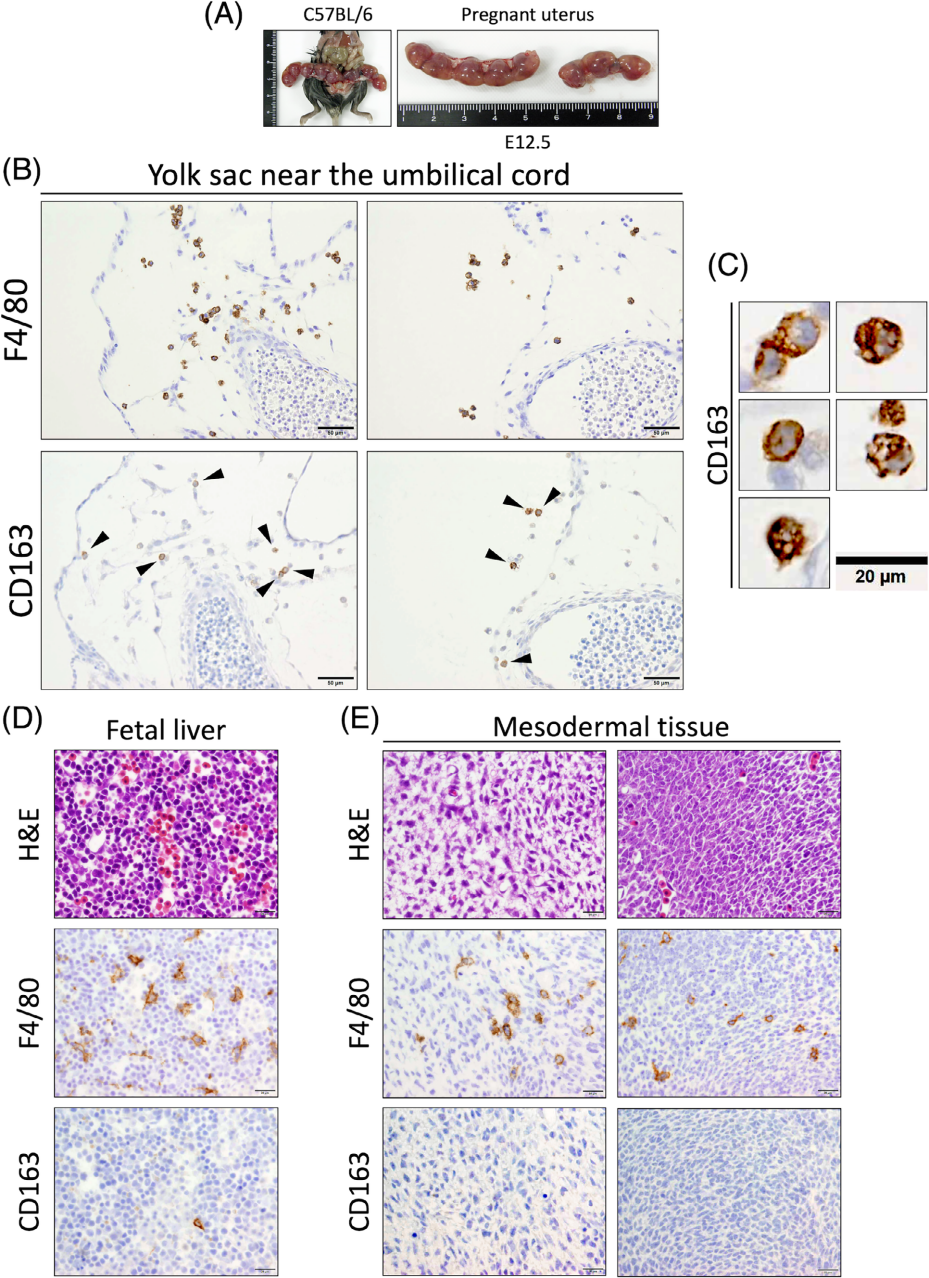
Localization of CD163‐positive macrophages at embryonic day (E) 12.5. (A) Macroscopic image of the pregnant mouse and uterus. Scale units: Cm. (B) Immunohistochemical staining of F4/80 and CD163 in the yolk sac near the umbilical cord. Arrowheads indicate CD163‐positive macrophages. Scale bars: 50 μm. (C) High magnification images of the representative CD163‐positive macrophages in B. Scale bar: 20 μm. (D,E) H&E and immunohistochemical staining of F4/80 and CD163 in the fetal liver (D) and the mesodermal tissue of the trunk (E). Scale bars: 20 μm.

Given the distribution of CD163‐positive macrophages in adult mice, we next analyzed their distribution in fetal mice. Organs were immunostained for CD163 at different embryonic days (E). As shown in Figure 15, the degree of localization of CD163‐positive macrophages was different in different organs and at different embryonal ages. CD163‐positive macrophages were first observed in the yolk sac at E12.5 (Figure 16A–C). During the same period, a small population of CD163‐positive macrophages was also observed in the blood sinuses of the liver and in the surface areas of the brain (Figure 16D). However, although F4/80‐positive macrophages were present, CD163‐positive macrophages were not observed in the majority of organs (Figure 16E). The distribution of CD163‐positive macrophages gradually expanded to various other organs after E12.5 (Figure 14). These findings suggest that CD163 is expressed only on a subset of yolk sac‐derived macrophages and not on MDMs in mice.

### 2.10 CD163 expression in macrophages of other primates and rodents

**FIGURE 17:**
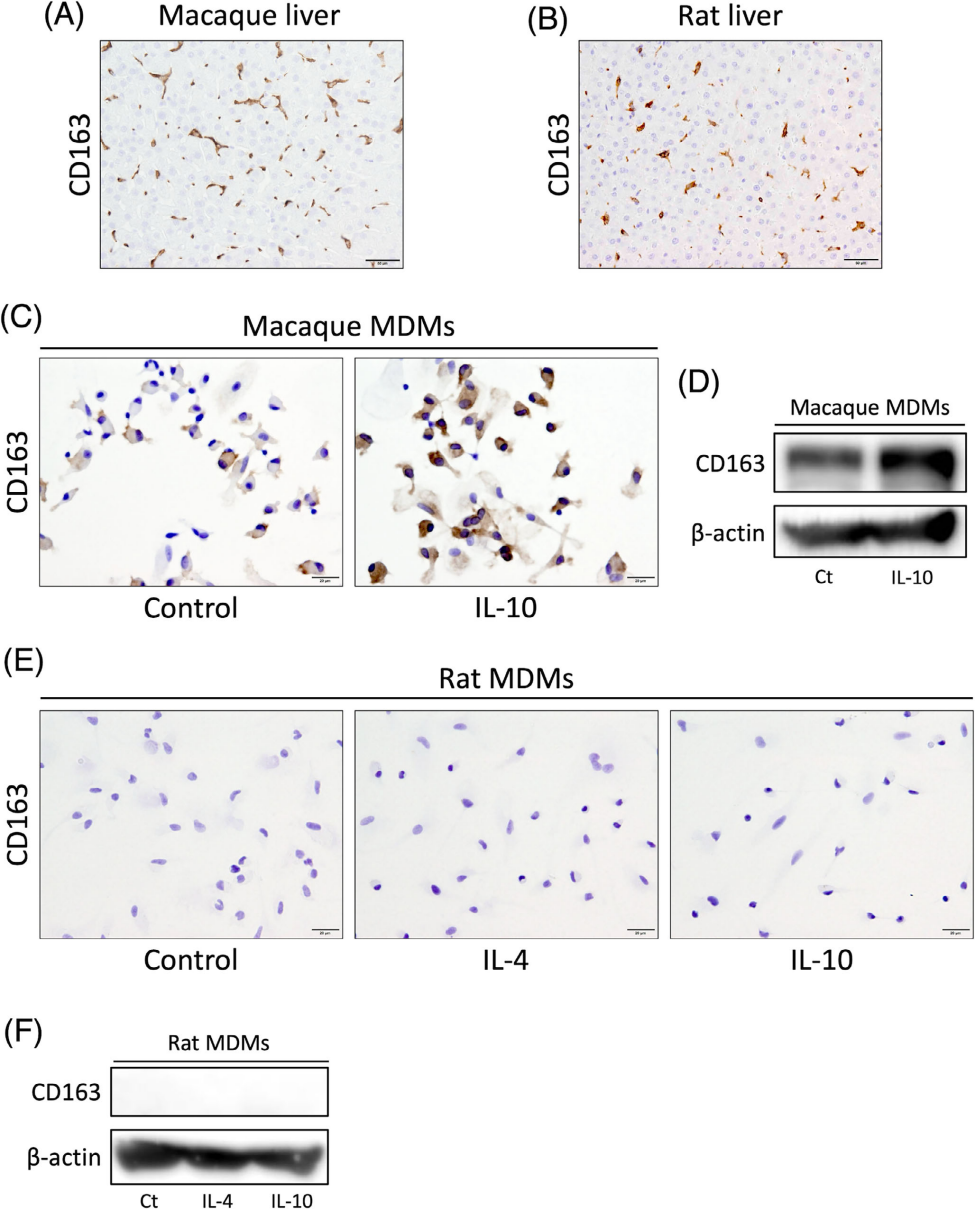
Differential CD163 expression between macaque and rat macrophages. (A,B) Immunohistochemical staining of CD163 in healthy macaque (A) and rat (B) liver. Scale bars: 50 μm. (C)–(F) Immunocytochemical staining (C, E) and western blotting (D, F) of CD163 in macaque (C, D) and rat (E, F) MDMs stimulated with IL‐4 or IL‐10 in the last 24 h of culture compared with untreated control macrophages. Scale bars: 20 μm.

To confirm whether these phenotypes were common to the respective order, that is, primates and rodents, we analyzed macaque and rat macrophages. CD163 was expressed on Kupffer cells from crab‐eating macaques and rats (Figure 17A,B). Similar to that observed with hMDMs (Figure 3A,C), CD163 expression was enhanced by IL‐10 stimulation in macaque MDMs (Figure 17C,D). In contrast, rat MDMs did not express CD163 even on stimulation with IL‐4 or IL‐10 (Figure 17E,F), consistent with mMDMs (Figure 3G).

**FIGURE 18:**
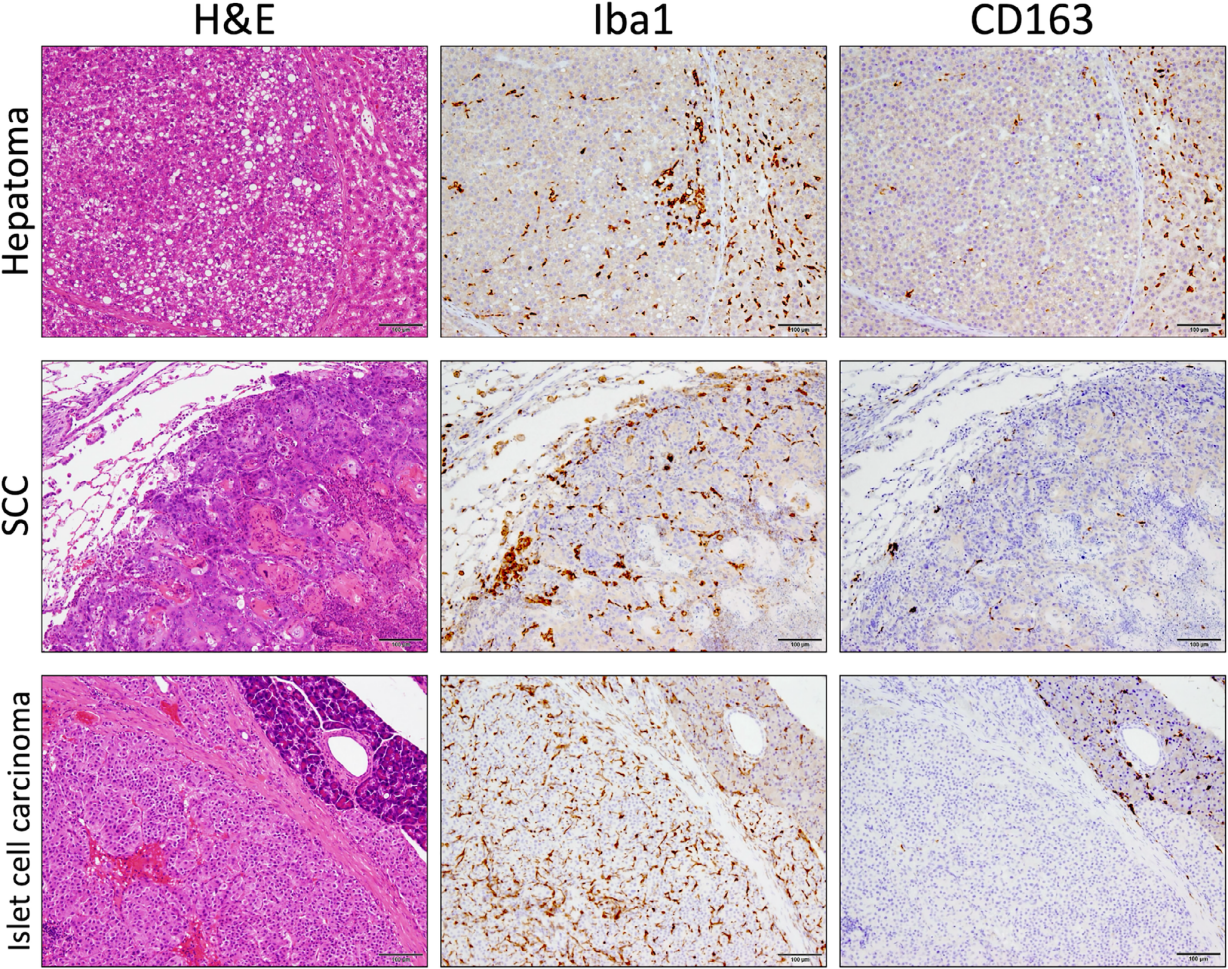
CD163 expression in spontaneous rat tumors. H&E and immunohistochemical staining of Iba1 and CD163 in hepatoma, squamous cell carcinoma (SCC), and islet cell carcinoma. Scale bars: 100 μm.

Furthermore, rat spontaneous tumors available in our lab, including hepatoma, lung squamous cell carcinoma, and pancreatic islet cell carcinoma, were immunostained for Iba1 and CD163 (Figure 18). We found that while Iba1^+^ TAMs infiltrated the entire tumor, CD163^+^ TAMs were almost absent in the tumor. This histology was very similar to that observed in mice (Figures 2A and 5D). Taken together, these findings suggest that rodent MDMs may not express CD163.

## 3 DISCUSSION

Although a few empirical reports suggest that TAMs infiltrating the center of tumors in mice do not express CD163, no studies have focused on this phenomenon.^24^, ^39^ The present study confirmed the absence of CD163 expression on central TAMs in both mouse and rat tumors using a variety of cell types and models (Figures 2, 5, 6, 7, and 18). Additionally, we confirmed that mouse and rat MDMs and mouse BMDMs do not express CD163 in vitro (Figures 3 and 17). Because too many sacrifices are required to establish sufficient MDMs in mouse, BMDMs were used as replacement cells for MDMs in most analyses except Figure 3G. BMDMs may be contaminated with small amounts of bone marrow‐resident macrophages (BMRMs) which are CD163‐positive as shown in Figure 14. Fischer‐Riepe et al. reported that differentiation of freshly prepared BM cells to classical BMDMs was accompanied by a strong reduction of CD163 expression due to a loss of a resident subpopulation of already differentiated macrophages in the BM (BMRMs) that are replaced during culture by macrophages developing from undifferentiated BM precursors (BMDMs).^40^ They also confirmed that CD163 cells represent only 1%–2% of all BM cells and CD11b^+^, F4/80^+^ BMDMs did not express CD163. Our results using BMDMs are consistent with their report. Furthermore, the observed absence of CD163 expression was replicated when macrophages were depleted using clodronate liposomes in vivo (Figures 10 and 11). Despite the limitation of a one‐month observation period, our findings confirm that in contrast to human macrophages, rodent MDMs do not express CD163. As shown in Figures 3G and 4C, the lack of responsiveness of mMDM to various cytokines in CD163 expression is unique. Thus, genetic modifications such as methylation of the promoter region might have affected this unique negative phenotype.

**FIGURE 19:**
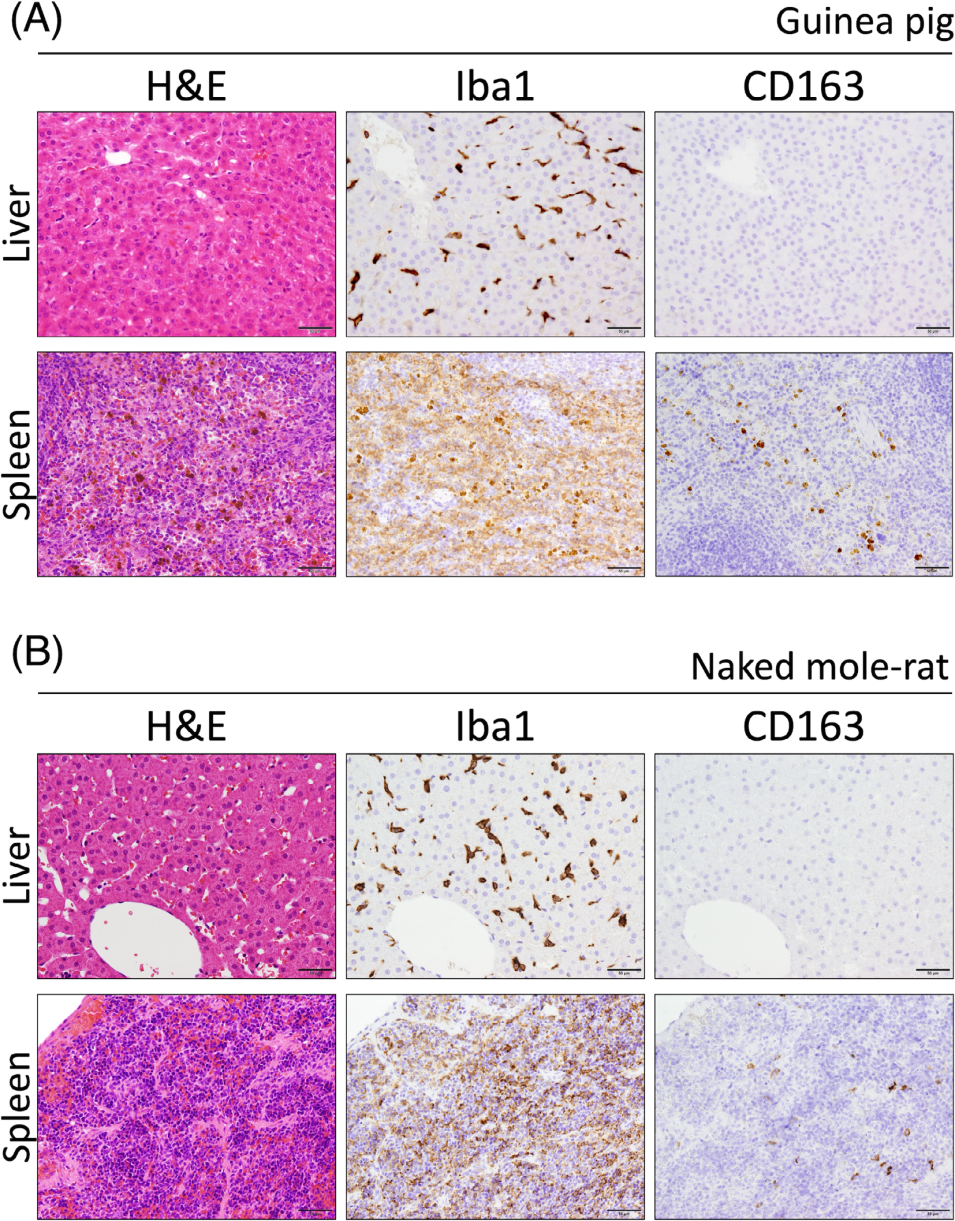
CD163 expression in the liver and spleen of Hystricomorpha species. (A,B) H&E and immunohistochemical staining of Iba1 and CD163 in the normal liver and spleen of guinea pig (A) and naked mole‐rat (B). Scale bars: 50 μm.

Mice and rats, classified within the Myomorpha suborder of rodents, were compared to guinea pigs and naked mole‐rats, which belong to the Hystricomorpha suborder of rodents, to determine if the entire rodent population exhibits a similar CD163 expression pattern. Organ specimens from guinea pigs and naked mole‐rats were analyzed for this purpose. CD163‐positive macrophages were notably fewer in the normal liver and spleen of Hystricomorpha species compared to that in Myomorpha species (Figure 19). Wada et al. reported that BMDMs of naked mole‐rats and their cell lines did not express CD163, despite expressing other common macrophage markers.^41^ These findings suggest that Hystricomorpha may have the same or more limited macrophage CD163 expression compared to Myomorpha, supporting the hypothesis that a mouse‐type CD163 expression pattern could be common across rodents.

**FIGURE 20:**
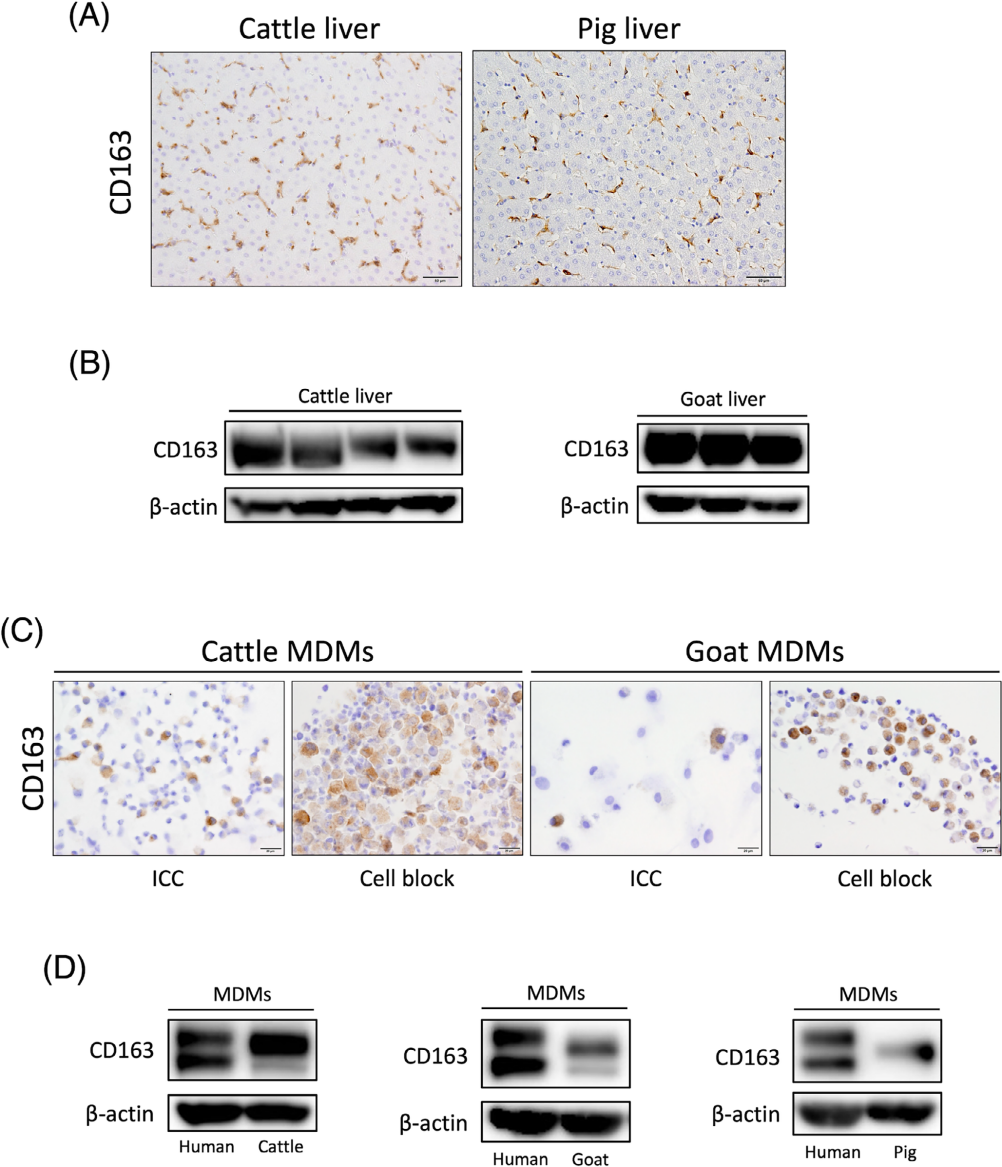
CD163 expression in the liver and MDMs of Cetartiodactyla species. (A) Immunohistochemical staining of CD163 in healthy cattle and pig liver. Scale bars: 50 μm. (B) Western blotting of CD163 in cattle and goat liver. (C) Immunocytochemical staining of CD163 in cattle and goat MDMs. Scale bars: 20 μm. (D) Western blotting of CD163 in cattle, goat, and pig MDMs compared with hMDMs.

**FIGURE 21:**
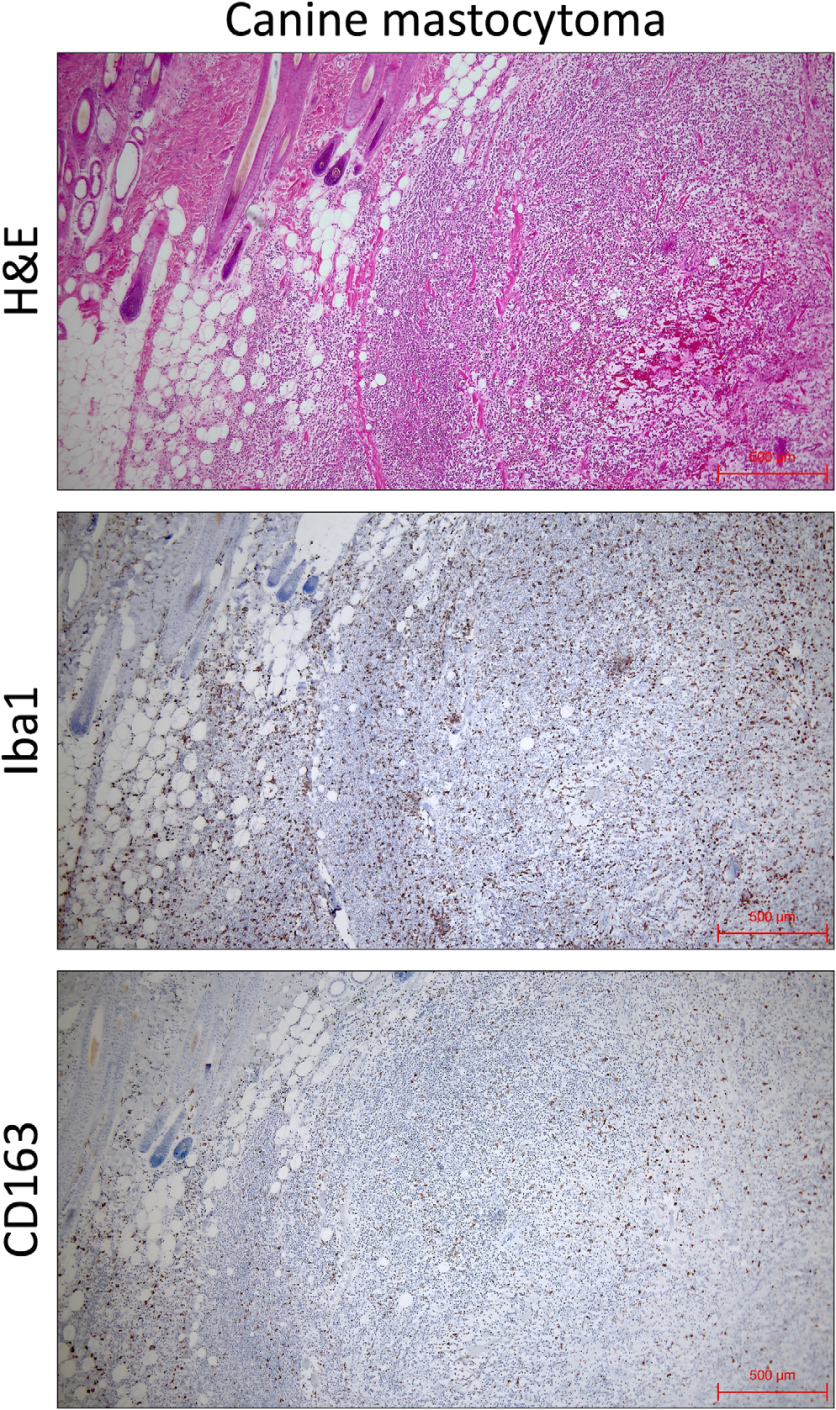
CD163 expression in canine subcutaneous mastocytoma. H&E and immunohistochemical staining of Iba1 and CD163 in canine subcutaneous mastocytoma. Scale bars: 500 μm.

Although CD163 has been studied in a variety of mammals, including domesticated and pet species, no reports have highlighted interspecies differences to date. We confirmed that MDMs from both humans and crab‐eating macaques, which are primate species, express CD163 (Figure 17). To determine whether CD163 expression in other mammalian macrophages resembles the pattern found in mice or humans, we collected liver and peripheral blood samples from livestock animals within the Cetartiodactyla order. The results are presented in Figure 20. CD163 expression was detected in the Kupffer cells of cattle, goats, and pigs through immunostaining and Western blotting (Figure 20A,B). Similarly, CD163‐positive MDMs were observed in these same species (Figure 20C,D). We previously reported an increase in CD163‐positive macrophages in the liver of a non‐alcoholic fatty liver disease (NAFLD) model of microminipigs, even in the presence of chronic inflammation.^42^ This finding is distinctly different from that observed in the mouse obesity model depicted in Figure 12. Monocytes in cattle, goats, sheep, pigs, and cetaceans have been reported to be CD163‐positive.^8^, ^43^, ^44^ Kupffer cells in cetaceans were also reported to be CD163‐positive, with an observed increase in the number of CD163‐positive cells in specific liver infections.^45^, ^46^ Thus, CD163 expression in Cetartiodactyla macrophages is thought to be similar to that in humans. Furthermore, CD163‐positive TAMs were observed to spread throughout mast cell tumors, a common skin and subcutaneous tumor in canines (Figure 21). Previous reports of tumors in dogs and cats have presented histological images of CD163‐positive TAMs distributed within the tumors.^27^, ^28^, ^29^, ^47^ In some of these reports, the density of CD163‐positive TAM infiltration was positively correlated with tumor grade, similar to that in humans. Thus, Carnivores, including dogs and cats, are likely to exhibit human‐type CD163 expression. Based on the above findings, we suggest that mouse‐type CD163 expression may be exclusive to the rodent clade among boreoeutherians (Table 2). However, the CD163 expression pattern in lagomorphs, the sister group of rodents, has not been analyzed. Many CD163 antibodies with excellent staining efficiency are rabbit antibodies, making histological analysis of rabbits challenging. Furthermore, Makishima et al. reported that many tissue macrophages were CD163‐negative in normal tissues of hedgehogs, classified as Eulipotyphla within Laurasiatheria, except for macrophages in the splenic red pulp and dermis.^48^ Hence, we cannot conclude that the mouse‐type CD163 expression is rodent‐specific, and many Boreoeutherians, especially lagomorphs and Eulipotyphla, need to be analyzed to confirm the same.

**TABLE 2:** CD163 expression in MDM, TAM, and Kupffer cells of animals.

Animals					CD163		
Magnorder	Superorder	Order	Suborder/infraorder	Species	MDM/Monocyte	TAM in the center	Kuppfer cell
Boreoeutheria	Euarchontoglires (Gr. III)	Rodentia	Histricomorpha	Naked mole rat	(−)	ND	±
				Guinea pig	ND	ND	±
			Myomorpha	Mouse	−	−	+
				Rat	−	−	+
		Primates	Haplorhini	Crab‐eating macaque	+	ND	+
				Human	+	+	+
	Laurasiatheria (Gr. IV)	Cetartiodactyla	Suina	Pig	+	ND	+
			Ruminantia	Cattle	+	ND	+
				Goat	+	ND	+
				Sheep	(+)	ND	ND
			Whippomorpha	Cetacean	(+)	ND	(+)
		Carnivora	Feliformia	Cat	ND	(+)	ND
			Caniformia	Dog	ND	+	ND
		Eulipotyphla		Hedgehog	ND	ND	(−)

*Note*: Data in brackets are from cited references.

Abbreviations: MDM, monocyte‐derived macrophage; ND, not done; TAM, tumor‐associated macrophage.

We were fortunate to be able to analyze a mouse specimen with overlapping hepatoma and histiocytic sarcoma. In contrast to TAMs in hepatoma, sarcoma cells were diffusely CD163‐positive (Figures 6 and 7). Because mMDMs, including TAMs, were CD163‐negative, we believe that the sarcoma cells were derived from CD163‐positive Kupffer cells. Janke et al. studied 62 cases of mouse histiocytic sarcomas and reported that “CD163 is the most common marker used to diagnose histiocytic sarcoma in humans, however, CD163 was expressed in only 34 of 61 (56%) mouse tumors”.^49^ They also presented histological images of focal CD163‐positive cells in a CD163‐negative tumor case, describing the foci as either reactive Kupffer cell hyperplasia or a subset of CD163‐positive tumor cells. When combined with our findings, we can hypothesize that CD163‐positive and CD163‐negative sarcoma cells may be derived from Kupffer cells and MDMs, respectively. Although human histiocytic sarcoma is rare, two reports investigating five cases found all cases to be strongly and diffusely positive for CD163 expression.^50^, ^51^ The higher frequency of CD163 positivity in humans may be attributed to the ability of hMDMs to express CD163. In one case report, a spontaneous histiocytic sarcoma in the liver of a hamster was also CD163‐positive.^52^ Histiocytic sarcoma in cats, dogs, ferrets, and hedgehogs has been reported to be both CD163‐positive and negative.^48^, ^53^, ^54^, ^55^, ^56^, ^57^, ^58^, ^59^ One case report each of orangutan and rabbit histiocytic sarcoma were CD163‐negative.^60^, ^61^ Notably, histiocytic sarcoma in redfin needlefish in Florida was reported to be CD163‐positive, based on several investigated cases.^62^ Based on expression frequency, CD204 has been reported to be more useful in diagnosing canine histiocytic sarcoma than CD163.^59^ In summary, CD163 is expressed in histiocytic sarcoma of various animals; however, it is difficult to discern a consistent pattern in expression frequency. At least in rodents, CD163‐positive histiocytic sarcomas are more likely to originate from CD163‐positive resident macrophages in the primary organ.

Based on the above results, we considered CD163 as a potential marker for a certain subset of resident macrophages in mice, and analyzed the organ distribution of CD163 from the fetus to adult stage in mice (Figures 14 and 15). Based on the assumption that CD163 is a marker for all resident macrophages, we noted a clear contradiction in the fact that alveolar macrophages, Langerhans cells, and microglia were CD163‐negative in adult mice (Table 1). Particularly, since microglia, which are presumably derived from yolk‐sac‐derived primitive macrophages, were CD163‐negative, CD163 cannot be a marker for this cell type.^63^, ^64^ On the other hand, CD163‐positive cells were first observed in significant numbers in the yolk sac around the umbilical cord on E12.5 (Figure 16). At the same time, a very small population of CD163‐positive cells was observed in the liver, where many F4/80‐positive cells were already present. Subsequently, CD163‐positive cells gradually spread to systemic organs (Figure 15). The distribution pattern of CD163‐positive cells during the fetal development of mice is reminiscent of fetal liver‐derived monocytes/macrophages. Although we attempted to generate CD163‐LacZ mice for further detailed histological analysis, the modification was lethal.

In light of the above findings, the previously reported findings using CD163KO mice are noteworthy. Differences between WT and CD163KO mice are only observed in resident macrophages such as large peritoneal macrophages or Kupffer cells. In other words, MDMs in both WT and CD163KO mice do not express CD163. For example, differences in tumor growth between WT and CD163KO mice reflect the functional differences of CD163 in resident macrophages in the subcutaneous area where tumor cells are implanted. The relatively greater importance of CD163 function in resident macrophages during the early stages of tumor cell engraftment may have led to significant differences in tumor growth and prognosis in our mouse sarcoma model.^24^ On the other hand, in humans, TAMs derived from hMDMs are M2‐activated by the tumor microenvironment and can express CD163.^18^, ^23^, ^24^ Since CD163 is not expressed in mMDMs of WT mice, there are likely to be significantly fewer reports on the correlation between CD163KO mice and tumor growth or prognosis. Etzerodt et al. reported selective depletion of CD163‐positive macrophages strongly inhibited tumor growth even in mouse.^39^, ^65^ The tumor regression was surprisingly caused by the recruitment and activation of tumor‐infiltrating lymphocytes, independent of anti‐PD‐1 treatment, in melanoma.^39^ In addition, a subset of CD163‐positive macrophages, which were reported to be derived from embryonic macrophages and self‐renewing, promoted the cancer stem cell‐like phenotype of ovarian cancer cells.^65^ These findings also suggest that the function of CD163‐positive resident macrophages, which are present from the early stage, is important for tumor growth. Most emphatically, CD163‐positive macrophages play a major role in promoting tumor growth, irrespective of the animal species or cell origin.

When assessing diseases in which CD163‐positive macrophages are known to be reduced, further caution is needed when comparing mouse and human models. Fatty liver induced by a HFD showed increased inflammatory cytokines and decreased CD163‐positive macrophages; however, this was observed only in mice (Figures 12 and 13). In the non‐rodent fatty liver disease, increased soluble CD163 in the blood was often reported, as well as increased CD163‐positive cells in the liver. The decrease in CD163‐positive cells in murine fatty liver can be simply explained by the infiltration of mMDMs that cannot express CD163. Nakashima et al. reported similar results in their analysis using flow cytometry.^38^ Since macrophages generally show M1 activation in inflammatory diseases, Kupffer cells may have decreased CD163 expression. Interestingly, however, they reported that although there was a clear downward trend of Kupffer cells in the high fat and cholesterol diet‐fed mice, there were no significant changes in between CD163‐positive and CD163‐negative Kupffer cells after high fat and cholesterol diet. Their results indicated that the CD163 expression rate of Kupffer cells is not significantly reduced in a similar fatty liver model. Hence, it should always be noted whether the reduction in CD163 expression is due to a reduction in CD163 expression in resident macrophages or a relative reduction due to infiltration of CD163‐negative mMDMs. Furthermore, when analyzing the correlation between inflammation and macrophage activation across mouse and human models, confirmation by markers other than CD163 is important. Still, the anti‐inflammatory M2 function of CD163 in non‐neoplastic inflammatory diseases might be common among rodent and other animals. There are reports that CD163KO mice exhibit increased sepsis mortality,^34^ enhanced collagen‐induced arthritis,^35^ and delayed fracture healing processes compared to WT mice.^36^ These findings suggest that CD163 expression in macrophages, other than mMDMs, contributes negatively to inflammation and is associated with better prognosis even in rodent non‐neoplastic inflammatory diseases.

In conclusion, in the present study, we found that mMDMs do not express CD163, which was confirmed by various methods. We also found that CD163‐positive macrophages in mice may originate from a subset of resident macrophages, specifically, fetal liver monocytes/macrophages. Furthermore, we revealed that the observed CD163‐negative expression pattern in MDMs is shared by the rodent clade. Given that CD163 is a conserved protein in many animals, it is important to elucidate both the molecular and cellular functions of CD163 across a wide variety of diseases and a broad range of animals, including humans, livestock, pets, and other mammals. Our findings regarding the specificity of the CD163 expression pattern in rodents indicate that more advanced functional research of CD163 and CD163‐positive macrophages among multiple animals using embryological, phylogenetic, and evolutionary approaches are needed.

## 4 EXPERIMENTAL PROCEDURES

### 4.1 Histological samples and staining

Formalin‐fixed (10% natural buffered) and paraffin‐embedded (FFPE) human specimens were obtained from patients with cancer who had undergone surgery at Kumamoto University Hospital. FFPE tumors, organs, and macrophage cell blocks of each animal were cut into 3 μm serial sections for hematoxylin and eosin staining or immunohistochemistry. Cultured macrophages on chamber slides were fixed with 10% natural buffered formalin for hematoxylin and eosin and immunocytochemistry. For antigen retrieval, the sections were immersed in EDTA solution (pH 8.0) (for CD163, CD68, and Iba‐1) or antigen retrieval solution (pH 9.0) (Nichirei, Tokyo, Japan) (for CD206) and heated in a pressure cooker or incubated with proteinase K at room temperature for 3 min (for F4/80). The following primary antibodies were used: anti‐CD163 antibodies (clone 10D6; Leica Biosystems, Nussloch, UK) (for human), anti‐CD163 polyclonal antibodies (ICA‐TG4‐RBP1; Cosmo Bio, Tokyo, Japan) (for all animals), anti‐CD163 antibodies (clone EPR19518; Abcam, Cambridge, UK) (for all animals), anti‐CD68 antibodies (clone PG‐M1; Agilent, Santa Clara, CA, USA) (for human), anti‐F4/80 antibodies (clone Cl:A3‐1; Bio‐Rad, Hercules, CA, USA) (for mouse), anti‐CD206 polyclonal antibodies (ab64693; Abcam) (for mouse), and anti‐Iba1 polyclonal antibodies (019‐19741; Fujifilm Wako, Osaka, Japan) (for rat, guinea pig, naked mole‐rat, and dog). An isotype‐matched mouse, rat, or rabbit IgG (Agilent) was used as a negative control. Following incubation with the primary antibodies at room temperature for 90 min, the samples were incubated with horseradish peroxidase‐labeled goat anti‐mouse, anti‐rat, or anti‐rabbit antibodies (Nichirei) at room temperature for 30 min. Immunoreactions were visualized using the diaminobenzidine (DAB) substrate system (Nichirei).

### 4.2 Mice and intervention models

C57BL/6, C3H, or BALB/c‐nu mice were purchased from CLEA Japan (Tokyo, Japan). Mice were kept in a temperature‐controlled house under specific pathogen‐free conditions with a 12‐h light/dark cycle. Subcutaneous tumor model: each strain of mice (8–10 weeks) was subcutaneously injected with OV2944 (C57BL/6×C3H), MCA205 (C57BL/6), LM8 (C3H), ED (BALB/C‐nu), or MC38 (C57BL/6) cells (5 × 10^5^) suspended in 50 μL of D‐MEM (Fujifilm Wako). Mice were sacrificed when the subcutaneous tumor grew to 10 mm in diameter. DMN‐induced hepatoma model: C57BL/6 mice were administered DMN (10.5 mg/kg) intraperitoneally at 8 and 15 days of age, followed by determination of DMN‐induced hepatoma development. Mice were sacrificed at 15 months of age. Clodronate liposome‐induced macrophage depletion model: C57BL/6 and C3H mice (8–10 weeks) administered clodronate liposome (Clophosome‐A, Funakoshi, Tokyo, Japan) (700 mg/kg) intraperitoneally for macrophage depletion. Mice were sacrificed on 3 days or 1 month post‐injection. HFD‐induced obesity model: C57BL/6 mice were fed an HFD (CLEA Japan) for 3 months. Mice were sacrificed after confirmation of obesity. Adult C57BL/6 mice and embryos were used for CD163 expression analysis using immunostaining.

### 4.3 Isolation and culture of hMDMs

RPMI 1640 (Fujifilm Wako) supplemented with 1% penicillin/streptomycin (Fujifilm Wako) and 10% FBS (Gibco, NY, USA) was used for the conditioned medium. Peripheral blood mononuclear cells (PBMCs) were isolated from peripheral blood obtained from healthy volunteer donors via density gradient centrifugation with Lymphoprep (Serumwerk, Bernburg, Germany). Monocytes were isolated from the PBMCs using a magnetic bead‐based isolation procedure (MACS CD14 microbeads, MACS column and separator; Miltenyi Biotec, Bergisch Gladbach, Germany). Isolated monocytes were cultured in polystyrene dishes (Becton Dickinson, NJ, USA) in the conditioned medium supplemented with macrophage colony‐stimulating factor (M‐CSF) (50 ng/mL) or granulocyte‐macrophage colony‐stimulating factor (GM‐CSF) (10 ng/mL) for 7 days to induce the differentiation of hMDMs.

### 4.4 Animal samples and isolation and culture of macrophages

Wistar rats were purchased from Kyudo (Kumamoto, Japan). The rats were housed in a temperature‐controlled rooms under a pathogen‐free barrier facility with a 12‐h light/dark cycle and were fed a normal rodent chow diet (CLEA Japan). Naked mole‐rats used in this study were raised in rooms at Kumamoto University that were maintained at 30 ± 0.5°C and 55% ± 5% humidity with a 12‐h light/dark cycle. Canine tumor specimens were provided by LST Inc. (Kumamoto, Japan). Peripheral blood samples and livers of healthy crab‐eating macaques, guinea pigs, and livestock were purchased from SNBL (Kagoshima, Japan), LST Inc., Swine Extension & Consulting Inc. (Niigata, Japan), or Kumamoto Livestock Distribution Center (Kumamoto, Japan), respectively. Animal PBMCs were isolated from peripheral blood via density gradient centrifugation with OptiPrep (Serumwerk). Bone marrow cells and PBMCs were cultured in polystyrene dishes (Becton Dickinson) in the conditioned medium supplemented with M‐CSF (50 ng/mL) for 7 days. Non‐adherent cells were removed to isolate BMDMs and MDMs, respectively. Peritoneal macrophages were obtained from peritoneal lavage using phosphate‐buffered saline.

### 4.5 Western blotting

Cells were solubilized with 1% NP‐40, and the proteins (5–30 μg) were electrophoresed on a 10% sodium dodecyl‐sulfate–polyacrylamide gel. Proteins were then transferred to polyvinylidene fluoride membranes (Millipore, Burlington, MA, USA). The membranes were cut and incubated with the following primary antibodies: anti‐CD163 antibodies (clone EPR19518; Abcam) (for all animals), anti‐CD204 antibodies (clone MCA1322GA; Bio‐Rad) (for mouse), anti‐CD206 antibodies (clone MCA2235GA; Bio‐Rad) (for mouse), and anti‐β‐actin antibodies (clone C4; Santa Cruz Biotechnology, Dallas, TX, USA) (for all animals). The membranes were then incubated with horseradish peroxidase‐conjugated anti‐mouse, anti‐rat, or anti‐rabbit IgG antibodies (Thermo Fisher Scientific, Waltham, MA, USA), and visualized with Pierce ECL Plus Western Blotting Substrate (Thermo Fisher Scientific). Signals were detected using an Amersham Imager 680 (GE Healthcare, Chicago, IL, USA).

### 4.6 Real‐time quantitative PCR

Total RNA was extracted with RNA iso‐Plus (Takara Bio, Shiga, Japan). The reverse transcription (RT) reaction was carried out using a PrimeScript RT reagent kit with gDNA Eraser (Takara Bio), and the following primers were used to amplify the target genes: human *CD163*: 5'‐CGAGTTAACGCCAGTAAGG‐3', 5'‐GAACATGTCACGCCAGC‐3'; human *β‐actin*: 5'‐ATTCCTATGTGGGCGACGAG‐3', 5'‐AAGGTGTGGTGCCAGATTTTC‐3'; mouse *CD163*: 5'‐TCTGGGGTGAAGAATTCCAG‐3', 5'‐GCCTGCCAGACGAATATCTATG‐3'; mouse *CCL2*: 5'‐CCAGCAAGATGATCCCAATG‐3', 5'‐TCTGGACCCATTCCTTCTTG‐3'; mouse *TNF‐α*: 5'‐CCA AAGGGATGAGAAGTTCC‐3', 5'‐TCCACTTGGTGGTTTGCTAC‐3'; mouse *IL‐6*: 5'‐AGTCCTTCCTACCCCAATTTCC‐3', 5'‐CTTGGTCCTTAGCCACTCCTTC‐3'; mouse *β‐actin*: 5'‐TTTCCAGCCTTCCTTCTTGG‐3', 5'‐TGGCATAGAGGTCTTTACGGATG‐3'. Quantitative PCR was performed using Applied Biosystems 7300 (Carlsbad, CA, USA). The expression of target genes was standardized to that of *β‐actin*.

### 4.7 Microarray

To induce the macrophage subtypes, the hMDMs and mBMDMs were stimulated for 24 h with lipopolysaccharide (10 ng/mL) + IFN‐γ (50 ng/mL); M1p, IFN‐γ (50 ng/mL); M1m, IL‐4 (10 ng/mL); M2a, IL‐1β (10 ng/mL); M2b and IL‐10 (10 ng/mL); M2c. Control M0 macrophages were incubated for 24 h without additional factors. Microarray analysis was performed as described previously.^66^ Signal values obtained in microarray experiments of hMDM and mBMDM subtypes were transformed to the log base 2, and the 75th percentile shift normalization was performed, which used the 75th percentile signal value as 0. The array has multiple probes assigned to one gene. The unique representative probe was selected using the highest median signal.

### 4.8 Statistical analysis

Statistical analyses were performed with Prism (GraphPad Software Inc., Boston, MA, USA). Data were expressed as the mean ± standard deviation. Two‐group comparisons were made using Student's *t*‐test or the Mann–Whitney *U*‐test. A *p* value of less than .05 was considered statistically significant.

## FUNDING INFORMATION

This study was supported in part by Grants‐in‐Aid for Scientific Research from the Ministry of Education, Culture, Sports, Science, and Technology of Japan (16H07079, 18J01441, 19K07459, 23K06362 and 22K04845).

## CONFLICT OF INTEREST STATEMENT

Yoshiyuki Hizukuri, Kyoko Yamashiro, and Yasuhiro Hayashi were employees of Asubio Pharma Co., Ltd. and are currently employees of Daiichi Sankyo Co., Ltd. The other authors have no potential conflicts of interest to declare.

## PATIENT CONSENT STATEMENT

All study participants provided written informed consent to participate in the study.

## Data Availability

The datasets analyzed during the study are available from the corresponding author on reasonable request. The microarray data for human macrophages are also available from Gene Expression Omnibus (GEO) (registration no. GSE85346).
